# Microbiome: The impact of the microbiota–gut–brain axis on endometriosis-associated symptoms: mechanisms and opportunities for personalised management strategies

**DOI:** 10.1530/RAF-23-0085

**Published:** 2024-06-05

**Authors:** Francesca Hearn-Yeates, Andrew W Horne, Siobhain M O’Mahony, Philippa T K Saunders

**Affiliations:** 1 EXPPECT Edinburgh and Centre for Reproductive Health, University of Edinburgh, Institute for Regeneration and Repair, Edinburgh, UK; 2 Department of Anatomy and Neuroscience, University College Cork, Cork, Ireland; 3 APC Microbiome Ireland, University College Cork, Cork, Ireland

**Keywords:** antibiotics, bloating, diet, dysbiosis, endometriosis, gut metabolome, gut microbiome, IBS, inflammation, microbiota–gut–brain axis, mood disorders, pain, probiotics, self-management strategies

## Abstract

**Graphical abstract:**

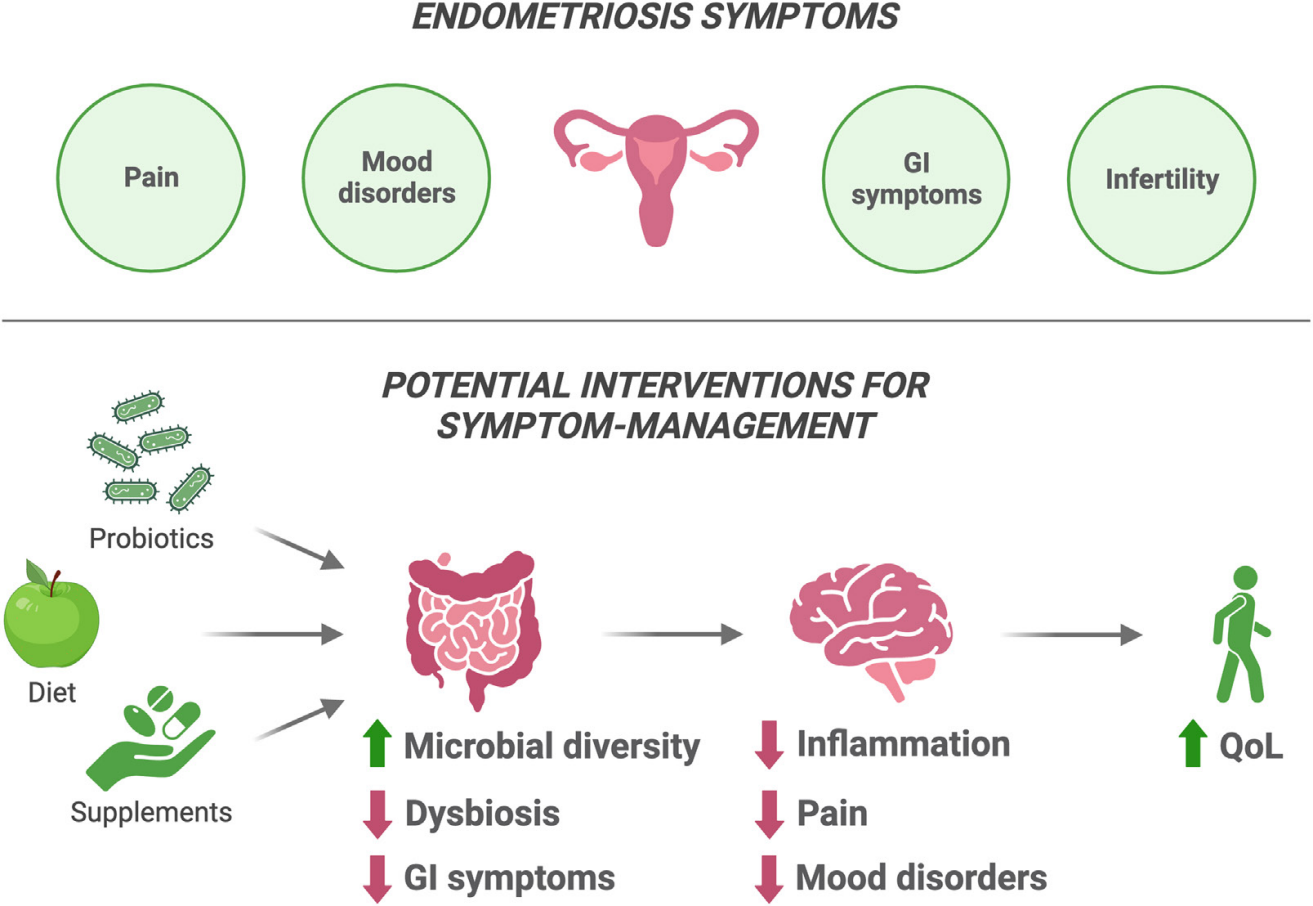

**Abstract:**

Endometriosis is a chronic inflammatory condition affecting one in ten women and those assigned female at birth, defined by the presence of endometrial-like tissue outside the uterus. It is commonly associated with pain, infertility, and mood disorders, and is often comorbid with other chronic pain conditions, such as irritable bowel syndrome. Recent research has identified a key role for the microbiota–gut–brain axis in health and a range of inflammatory and neurological disorders, prompting an exploration of its potential mechanistic role in endometriosis. Increased awareness of the impact of the gut microbiota within the patient community, combined with the often-detrimental side effects of current therapies, has motivated many to utilise self-management strategies, such as dietary modification and supplements, despite a lack of robust clinical evidence. Current research has characterised the gut microbiota in endometriosis patients and animal models. However, small cohorts and differing methodology have resulted in little consensus on the data. In this narrative review, we summarise research studies that have investigated the role of gut microbiota and their metabolic products in the development and progression of endometriosis lesions, before summarising insights from research into co-morbid conditions and discussing the reported impact of self-management strategies on symptoms of endometriosis. Finally, we suggest ways in which this promising field of research could be expanded to explore the role of specific bacteria, improve access to ‘microbial’ phenotyping, and develop personalised patient advice for reduction of symptoms such as chronic pain and bloating.

**Lay Summary**

Endometriosis is a chronic condition affecting one in ten women and those assigned female at birth, defined by the presence of tissue, similar to the womb lining, growing outside the womb. Symptoms include pelvic pain, period pain, pain during sex and when going to the toilet, digestive disturbance and bloating, infertility, depression, and anxiety. Standard treatments, including surgery and hormone-altering drugs, often have negative side effects. Many women with endometriosis use self-management strategies to control their symptoms, including changing their diet or taking supplements. Although some reports suggest such strategies are helpful, there is limited high-quality evidence to support their use. Here, we discuss how dietary adaptations could be impacting endometriosis-associated symptoms via changes to the bacteria within the gut. Gut bacteria communicate with the brain and influence inflammation throughout the body. Therefore, altering the gut bacteria through dietary changes can potentially benefit a variety of endometriosis-associated symptoms.

## Introduction

Endometriosis is a chronic inflammatory pain condition believed to impact the lives of one in ten women and those assigned female at birth (Horne & Missmer 2022). Whilst endometriosis is defined by the presence of endometrial-like tissue growing as ‘lesions’ outside the uterus, patients can present with a range of seemingly unrelated symptoms, leading to new, sometimes controversial, reframing of endometriosis as a body-wide disorder (Hickey *et al.* 2020). For example, whilst pain (cyclical or constant) and infertility are common symptoms, patients with endometriosis often present at clinics reporting a range of other problems. These include mood disorders (anxiety, depression) and symptoms affecting their digestive system, such as abdominal bloating and those mirroring irritable bowel syndrome (IBS) (Saunders & Horne 2021, Saunders & Horne 2023).

The gut microbiota is the collection of bacteria, viruses, and archaea within the gastrointestinal (GI) tract which produce essential metabolites, hormones, and neurotransmitters. Evidence for the impact of diet on the gut microbiota and the importance of the microbiome to general health is rapidly expanding (Cryan *et al.* 2019). Notably, metabolic products of the microbiota can affect the immune system and influence inflammation, leading to increased interest in how changes in the microbiota could impact on the severity of symptoms in disorders associated with aberrant immune responses, such as endometriosis (Saunders & Horne 2021). A breakthrough in our understanding of the importance of the bidirectionality in signalling between the gut and brain has been informed by results of studies on symptoms including stress, pain, and mood disorders (Rea *et al.* 2019, Wilmes *et al.* 2021). Studies such as these have linked gut dysbiosis (an ‘imbalance’ in the gut microbial community) to the severity of symptoms and vice versa.

Research into the relationship between the gut microbiota and endometriosis is still limited in scope, with a focus on endometriotic lesion development and disease progression, rather than its potential influence on symptomology (Chadchan *et al.* 2023, Wei *et al.* 2023). In this narrative review, we will provide a brief overview of the symptoms of endometriosis that may be altered by signalling within the microbiota–gut–brain (MGB) axis, briefly consider the existing primary research exploring the function of the gut microbiota in endometriosis lesion development, which has been explored in depth elsewhere (Talwar *et al.* 2022, Chadchan *et al.* 2023), before focussing on the potential role of dialogue between the gut microbiota, inflammatory response, and pain pathways in promoting/mitigating the body-wide symptoms associated with the disorder. To increase the range of evidence we will summarise findings from other chronic inflammatory pain conditions, to demonstrate the potential mechanisms of interaction between the gut microbiota and key symptoms of endometriosis: pain and inflammation; GI symptoms; and mood disorders. Finally, we will discuss promising therapeutic opportunities, such as dietary intervention, supplements, probiotics, and antibiotics, to alleviate symptoms via manipulation of the gut microbiota, providing exciting opportunities for future research with the priority of improving symptomology and patients’ quality of life (QoL), some of which have been conducted in cohorts of endometriosis patients.

Notably, as our understanding of the role(s) of other microbiomes has increased, researchers have also begun to explore whether the vaginal, endometrial, oral, and peritoneal microbiomes are altered in endometriosis patients, but results to date are variable. For the purposes of the current narrative review, we have focussed on the evidence that the gut microbiome, acting as part of a gut–brain bidirectional signalling system, can impact on symptoms of endometriosis, as well as evidence from studies on endometriosis patients and other disorders often co-morbid with endometriosis, that regulation of the microbiome could be a target for symptom relief.

### Search method

A comprehensive literature review identified articles and reviews through PubMed by searching for specific keywords including endometriosis, (chronic) pain, gut microbiome/metabolites, diet, supplements, IBS, mood, and other relevant related terms.

## Endometriosis

### Aetiology and pathogenesis

The exact cause of endometriosis is currently undetermined, although evidence shows genetic changes may increase the risk of developing the disorder (Zondervan *et al.* 2018, Saunders 2022). A defining hallmark of endometriosis is considered the presence of ‘lesions’ resembling endometrial tissue, most commonly detected in the peritoneal cavity (Saunders & Horne 2021). Our understanding of the mechanisms resulting in the establishment and survival of lesions has evolved from the theory of retrograde menstruation – the concept of menstrual debris entering the pelvic cavity via the Fallopian tubes during menstruation and implanting into the peritoneum, complemented by other routes including transfer via the vasculature (Yovich *et al.* 2020). In the past 20 years, evaluation of clinical samples and preclinical models have provided evidence to support a role for steroid hormone regulation of cell proliferation, inflammation, and neuroangiogenesis, with nerve projections connecting the lesions to the central nervous system (CNS), promoting the survival of lesion tissue and the development of pain symptoms (extensively reviewed elsewhere) (Zondervan *et al.* 2018, Zondervan *et al.* 2020, Saunders & Horne 2021).

Current therapeutic options for people with endometriosis are limited (Saunders & Horne 2021). Surgical removal of lesions, hormonal therapies, and analgesics are the most common strategies, all of which can be associated with detrimental side effects, with over 50% of patients having repeat surgeries within 5 years (Saraswat *et al.* 2018). Many endometriosis patients have reported trialling a selection of self-management strategies, including dietary interventions and dietary supplements, likely in response to the combination of diagnostic delays and the narrow range of therapeutic options currently available.

### Immune response and inflammation

An altered immune response and changes in immune-cell phenotype are reported in patients with endometriosis. Table 1 summarises endometriosis-associated changes in key immune cells. For example, several studies have shown increased infiltration of neutrophils and macrophages in the peritoneal fluid and lesions, with altered M1–M2 macrophage polarisation, alongside suppressed natural killer (NK) cell activity and increased numbers of T helper 17 cells (Th17) (Symons *et al.* 2018). Importantly, these changes are associated with increased levels of proinflammatory cytokines in the lesion microenvironment, whose downstream effects include increased inflammation, angiogenesis, and cell proliferation, all of which contribute to survival/growth of lesions (Herington *et al.* 2011, Symons *et al.* 2018). Studies such as these have supported the argument that endometriosis should be considered as an inflammatory disorder, and therapies sought to blunt/normalise these responses (see Saunders & Horne 2021).

**Table 1:** Summary of immune-cell changes in peritoneal fluid of endometriosis patients.

Immune cell type	Cellular changes	Cytokine and chemokine production	Downstream effects	Reference
Neutrophils	↑ PF infiltration; ↓ Apoptosis	↑ TNF-α; ↑ VEGF	↑ Inflammation; ↑ Angiogenesis	Symons *et al.* (2018)
Macrophages	↑ Infiltration in PF and lesion microenvironment; ↓ Phagocytosis; ↑ Activation of transcription factor NF-κB; Co-localisation with nerve fibres; altered M1–M2 polarisation	↑ IL-1β; ↑ IL-6; ↑ IL-10; ↑ TNF-α; ↑ TGF-β; ↑VEGF	↑ Inflammation; ↑ Angiogenesis; ↑ Stromal cell proliferation and invasiveness	Herington *et al.* (2011), Symons *et al.* (2018)
NK cells	↓ Activity	↑ TNF-α	↓ Cytotoxicity; ↑ Inflammation	Herington *et al.* (2011), Symons *et al.* (2018)
T cells	↑ Th17:Treg ratio	↑ IL-17 leads to: ↑ IL-8 and ↑ COX-2	↑ Inflammation; ↑ Angiogenesis; ↑ Stromal cell proliferation; attracts and activates neutrophils	Symons *et al.* (2018)

NK, natural killer.

### Symptoms which may be relevant to regulation by the MGB axis

#### Pain

**Figure 1:**
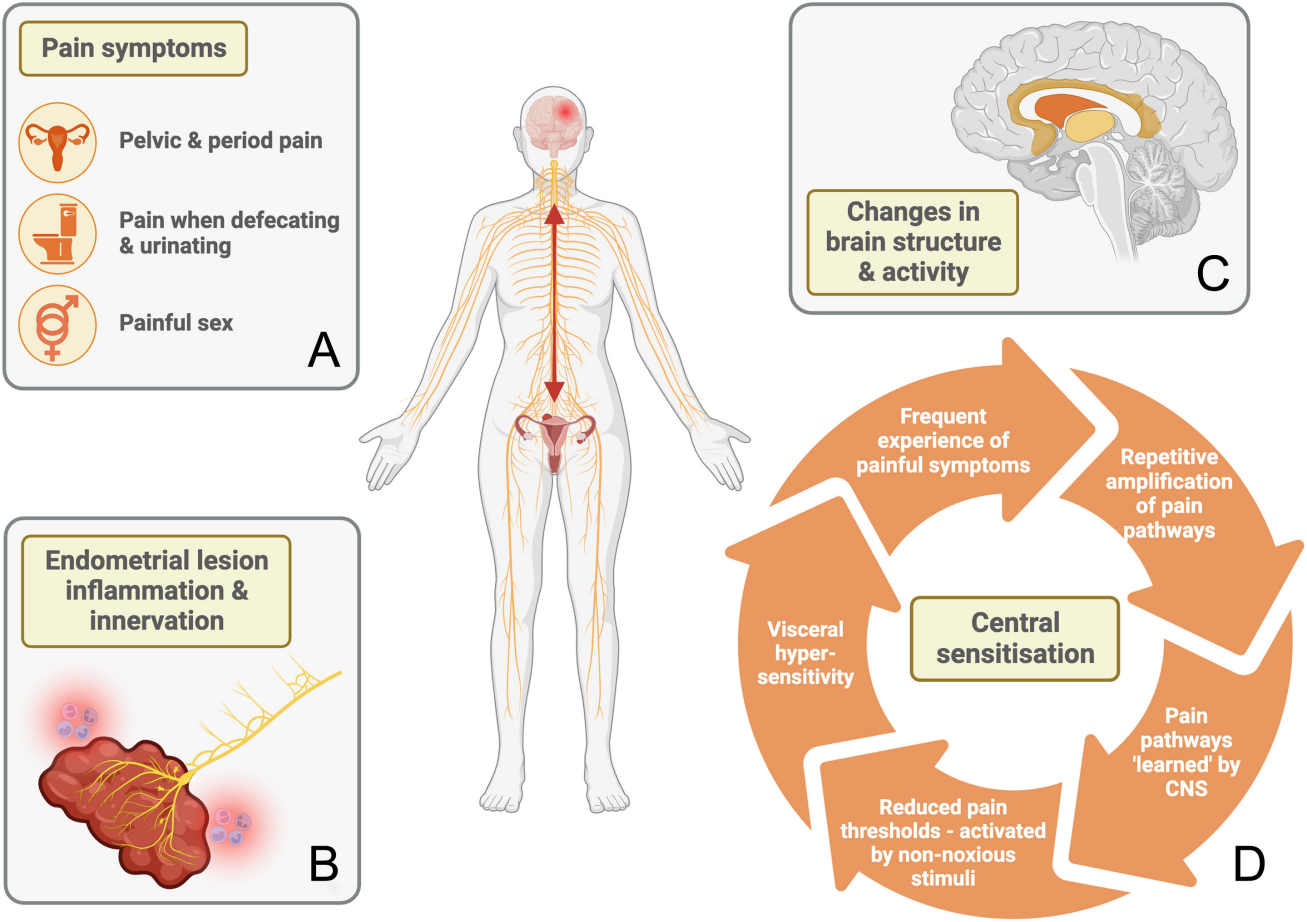
Pain mechanisms in endometriosis**.**A global assessment of pain mechanisms that may differ in endometriosis patients has identified alternations in chronic pain pathways associated with hypersensitivity to stimuli. (A) Commonly reported pain symptoms in endometriosis include dysmenorrhea (pain during menstruation), dyspareunia (pain during sex), and pain on defecation and urination (Saunders and Horne, 2021). (B) Inflammation and innervation of endometrial lesions contribute to pain experience via a connection with the CNS and recruitment of immune cells (Tokushige *et al.* 2010, Liu *et al.* 2012). (C) Differences have also been identified in the brains of endometriosis patients, with reduced grey matter volume in the left thalamus (pale yellow), right putamen (orange), left cingulate gyrus (dark yellow), and right insula of those with chronic pelvic pain (CPP) (As-Sanie *et al.* 2012), as well as changes identified in the extent and location of activation in response to painful stimuli in those with dysmenorrhea (Tu *et al.* 2009). (D) Central sensitisation occurs when pain pathways are persistently amplified, enabling them to be ‘learnt’ by the CNS, thus reducing the level of stimuli required to trigger pain, eventually leading to sensitivity to non-noxious stimuli and at sites distal to the inflammation (Berkley *et al.* 2005, Neziri *et al.* 2010). In CPP, this manifests as visceral hypersensitivity (Vincent *et al.* 2011, Kaya *et al.* 2013) and has been demonstrated in endometriosis (Weiwei *et al.* 2010, Aredo *et al.* 2017, Zheng *et al.* 2019). The red arrow represents the bidirectional relationship between pain pathways triggered by local inflammation and immune responses in the peritoneum (or alternative lesion locations) and the structural and learnt changes in the brain which result in exacerbated pain experiences. Created with BioRender.com.

Individuals with endometriosis report a variety of different types of pain (Fig. 1A). Mechanisms that initiate or promote endometriosis-associated pain symptoms remain the subject of intense research activity, with some evidence suggesting the role of nerve growth within lesions, which may occur in parallel with angiogenesis (Asante & Taylor 2011). Many studies have reported a lack of correlation between pain intensity and the number, location, or type of lesions (Vercellini *et al.* 2007), indicating other mechanisms also contribute to pain experience in addition to the extent/presence of lesion neurogenesis (Fig. 1B).

Reduced pain thresholds, along with increased activity in several brain regions associated with pain perception, have been identified in both cynomolgus monkeys with naturally occurring endometriosis (Yano *et al.* 2019) and a rat model of the disease (Zheng *et al.* 2020). Coupled with the fact that the diagnostic delay experienced by individuals with endometriosis may increase the likelihood of developing chronic/persistent pain that is resistant to standard therapies, it is clear that new approaches to pain management are required. In women with chronic pelvic pain (CPP), including those with endometriosis, changes in brain structures have been detected that appear consistent with an amplified/abnormal response to stimuli (Fig. 1C) (Brawn *et al.* 2014). Additional data have detected altered brain chemistry associated with these physical changes, consistent with amplification of pain signals and so-called central sensitisation augmenting signals from peripheral enteric nerves (Fig. 1D) (As-Sanie *et al.* 2016).

#### Symptoms associated with the GI system

There is increased awareness of the impact of GI symptoms on the wellbeing of endometriosis patients (Maroun *et al.* 2009). A large study found women with endometriosis had increased risk of inflammatory bowel disease, Crohn’s disease, and ulcerative colitis (Jess *et al.* 2012). A two-fold higher incidence of IBS in individuals with endometriosis, compared to the general population (Chiaffarino *et al.* 2021, Aupetit *et al.* 2022), suggests overlapping mechanisms between the conditions, recently corroborated by evidence of shared genetic risk factors (Yang *et al.* 2023). IBS is characterised by chronic gut inflammation, bloating, and visceral pain – symptoms common in patients with a diagnosis of endometriosis (Saunders & Horne 2021, Deepak Kumar *et al.* 2023). Crucially, patients highlight abdominal bloating as an important topic of unmet need (Horne *et al.* 2017).

#### Mood disorders and stress response

Women with endometriosis have a higher incidence of psychiatric comorbidities and mood disorders, including depression and anxiety (Gete *et al.* 2023). Mechanisms to explain these comorbidities are likely to involve the hypothalamic–pituitary–adrenal (HPA) axis, a neuroendocrine signalling pathway with a critical role in hormone regulation (Oyola & Handa 2017), whose dysregulation is involved in mood disorders (Bao & Swaab 2019). When activated, the HPA axis causes the adrenal cortex to release the glucocorticoid, cortisol, making it the primary coordinator of the stress response. Chronic pain can lead to dysregulation of the HPA axis, and this has been demonstrated in a range of inflammatory conditions (Kuehl *et al.* 2010). A small study recently found an association between a dysregulated HPA axis and menstrual pain severity in endometriosis patients (Ortiz *et al.* 2020). In a study of 26 women with endometriosis and CPP, physical and psychological therapy normalised cortisol levels, reduced perceived stress, and improved physical functioning (Friggi Sebe Petrelluzzi *et al.* 2012). There is an increasing appreciation of the role of the stress response and cortisol in modulating the MGB axis at multiple levels, including gut function and composition of the gut microbiota. A recent comprehensive review on the connections between the HPA and MGB axis was published by Rusch *et al.* (2023).

## Impact of the gut microbiome on general health and pain perception

In the following section, to provide a framework for considering the role of the gut microbiome and its metabolites in endometriosis, we provide a brief overview and references to some recent relevant papers.

### Gut microbiota

The gut microbiota is the community of microorganisms, including bacteria, viruses, and archaea, residing in the GI tract. The bacterial community is dominated by the phyla Firmicutes and Bacteroidetes, which comprise approximately 90% of the total gut bacteria (Sommer & Bäckhed 2013), with Fusobacteriota and Verrucomicrobiota present in low abundance (Cryan *et al.* 2019). Human gut microbiota have been broadly grouped into separate enterotypes, depending on levels of three specific genera: Bacteroides, Prevotella, and Ruminococcus. Differing enterotypes are associated with the consumption of certain diets: the Bacteroides and Prevotella enterotypes are associated with high-fat/high-protein diets and high-carbohydrate diets, respectively (Cryan *et al.* 2019).

Bacterial diversity can be differentiated by α- and β-diversity indices, with the former focussed on diversity within a single sample and the latter comparing population diversity between different samples (Wagner *et al.* 2018). Increased diversity is generally considered to be associated with improved health outcomes (Valdes *et al.* 2018). The gut microbiota plays essential roles in promoting gut health, including maintaining intestinal barrier function, and priming and maintenance of the immune system. The bacterial community regulates and activates both peripheral and resident immune cells, either via direct contact or compounds secreted through the mucus layer and gut epithelium, as well as signalling to the brain via nerve stimulation.

### Microbiota–gut–brain axis

The MGB axis is a two-way communication pathway linking the CNS and gut bacteria. Gut bacteria produce an assortment of vital metabolites including: bile acids, short-chain fatty acids (SCFAs), hormones, and neurotransmitters; which can signal to the brain and regulate a range of functions throughout the body (Liu *et al.* 2022).

Signalling between cells in the gut and brain is mediated via neural pathways involving the vagus nerve and the enteric/parasympathetic nervous system, as well as immunological and hormonal factors, including those contributing to the HPA axis (see the comprehensive review by (Cryan *et al.* 2019)). Briefly, associations have been found between altered parasympathetic nerve activity, pain, and bacterial composition, including evidence from CNS disorders (Wang & Kasper 2014). The production and utilisation of metabolic products, such as tryptophan and serotonin, by certain gut bacteria provide a secondary mechanism for their role in mood disorders via activation of the HPA axis (O’Mahony *et al.* 2015).

Gut microbiota also play a role in the maturation and maintenance of microglia, CNS-resident immune cells, which function in neuroinflammation and pain processing (Erny *et al.* 2015). Activation of these neuroimmune cells is considered one of the key mechanisms in central sensitisation due to the production of proinflammatory mediators, including IL-1β, interferon-γ, and TNF-α (Guo *et al.* 2019). These, amongst other cytokines and chemokines, disrupt the ratio of glutamate versus γ-aminobutyric acid (GABA) in synaptic transmission, leading to decreased pain thresholds (Ustianowska *et al.* 2022).

#### Short-chain fatty acids

SCFAs are an important product of bacterial metabolism, produced by certain bacterial species as a by-product of dietary fibre fermentation. They modulate the inflammatory status of the gut by regulating the immune response (Liu *et al.* 2022) and maintain the mucosal barrier by promoting the proliferation of intestinal epithelial cells (Vinolo *et al.* 2011). SCFAs act via two primary mechanisms: activation of G-protein-coupled receptors (GPCRs), GPR41 and GPR43, expressed on neutrophils and monocytes, and throughout the GI tract; and inhibition of histone deacetylases (Tan *et al.* 2014). SCFAs can promote peripheral Treg generation (Arpaia *et al.* 2013) and have been found to regulate neuroinflammation via the GPCR HCAR2 (Boccella *et al.* 2019), expressed during pain in the hypothalamus (Li *et al.* 2020).

#### Estrobolome

**Figure 2:**
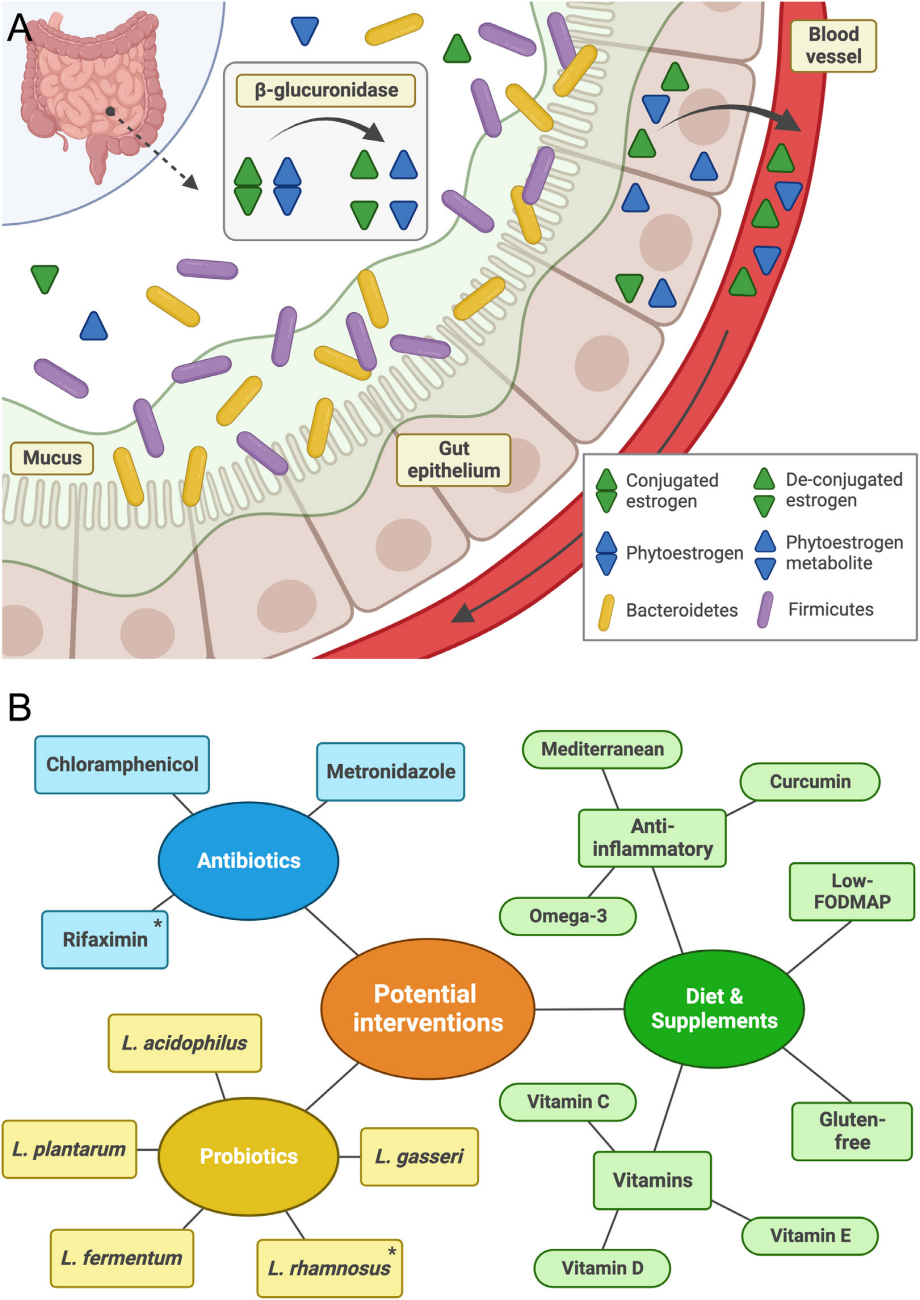
(A) The estrobolome.Certain gut bacteria, including Bacteroides, Bifidobacterium, Escherichia, and Lactobacillus, are capable of impacting circulating oestrogen concentrations. These bacterial genera have β-glucuronidase and β-glucosidase activity, enzymes that deconjugate endogenous oestrogen and exogenous phytoestrogens in the gut (Kwa *et al.*, 2016). A higher prevalence of these bacteria, or increased activity of the enzymes, leads to increased concentration of biologically active oestrogen metabolites in circulation, which may influence cell proliferation or immune responses (Symons *et al.*, 2018). On the other hand, decreased activity will diminish circulating free oestrogen leading to increased excretion of conjugated parent oestrogens, oestrone (E1) and oestradiol (E2) (Spichak *et al.*, 2018). Created with BioRender.com. (B) Summary of potential symptom-management strategies via manipulation of the gut microbiome.A broad range of interventions have been trialled in endometriosis patients for their potential beneficial impact on symptoms. Different dietary modifications have been the most extensively researched strategies, with antibiotic and probiotic treatments currently in their infancy. *Trials conducted only in patients with IBS not endometriosis. Created with BioRender.com.

The estrobolome is the collection of gut bacteria capable of altering the concentrations of bioactive steroids, including oestrogens, by enzymatic activities that cleave side chains from conjugated steroids (Fig. 2A). Examples include metabolism of oestrone-3-glucuronide and oestradiol-17-glucuronide, to oestrone (E1) and oestradiol (E2), respectively (Ervin *et al.* 2019). This has been further evidenced by a correlation between microbial diversity and higher E2 levels (Shin *et al.* 2019), with the bidirectionality of this relationship shown by the microbiota changes caused by ovariectomy (O'Mahony *et al.* 2017).

#### Dysbiosis and gut permeability

Gut dysbiosis, resulting from disturbances in normal microbiota communities, can be caused by many factors, such as stress, physical illness, antibiotics, and dietary changes (Valdes *et al.* 2018). Dysbiosis and inflammation can increase permeability of the intestinal barrier via weakening of the tight junctions, allowing movement of bacteria and pathogenic-associated molecular patterns (PAMPs) into circulation (Gieryńska *et al.* 2022). Subsequent recognition of PAMPs by TLRs triggers proinflammatory cytokine production, inducing activation of transcription factors, such as NF-κB (Kawai & Akira 2010). The downstream effects of these pathways result in both local and systemic low-grade inflammation.

## The gut microbiota and metabolites in endometriosis

### Research investigating the role of the gut microbiota and metabolites on disease progression

Animal models have been developed to simulate aspects of the aetiology and symptomology of endometriosis, including some using behavioural endpoints as a surrogate for pain (Tejada *et al.* 2023). The impact of the gut microbiota has been investigated using rodent models, complemented by studies in primates with naturally occurring endometriosis.

To date, the majority of studies have focussed on characterisation of gut microbiota following artificial induction of endometriosis (Table 2A). Unfortunately, the results reported in the different studies were not consistent, potentially due to the lack of standardised methods and outcome measures. Some studies reported positive effects of antibiotics (Chadchan *et al.* 2019), n-butyrate (Chadchan *et al.* 2021), or alpha-linolenic acid (ALA) (Ni *et al.* 2021), but no behavioural measurements were included, meaning it is impossible to assess whether there was any impact on pain.

**Table 2:** Studies in animal models (A) and human studies (B) investigating the role of gut microbiota and metabolites on endometriosis disease progression.

	**Findings**	**Reference**
A) Animal models		
Rhesus monkeys with naturally occurring endometriosis	- Higher concentrations of gram-negative bacteria	Bailey & Coe (2002)
	- Elevated levels of intestinal inflammation	
Endometriotic mouse model – i.p. injection	Findings 42 days after induction:	Yuan *et al.* (2018)
	- No significant differences in gut microbiota α-diversity	
	- Higher gut microbiota β-diversity	
	- Dysbiosis led to enriched Firmicutes	
Endometriotic mouse model – surgical	Findings 21 days after induction:	Chadchan *et al.* (2019)
	- Significantly lower gut microbiota α-diversity	
	- Higher abundance of Bacteroidetes and lower abundance of Firmicutes	
	- Microbiota depletion (MD) using broad-spectrum antibiotics significantly decreased the size of endometriotic lesions and the number of proliferative cells associated with a decrease in inflammatory markers	
	- MD followed by oral gavage with faeces from endometriotic, but not vehicle-treated, mice re-established lesion growth and inflammation	
Endometriotic mouse model – surgical	- No significant differences in gut microbiota α- or β-diversities after 7 and 21 days	Hantschel *et al.* (2019)
Endometriotic rat model – surgical	Findings 28 days after induction:	Cao *et al.* (2020)
	- Significantly lower gut microbiota α-diversity	
	- Higher abundance of Firmicutes and lower abundance of Bacteroidetes and Proteobacteria	
	- Gut microbiota β-diversity showed significant differences in species composition	
Endometriotic mouse model – i.p. injection	Findings 21 days after induction:	Ni *et al.* (2020)
	- Significantly lower gut microbiota α-diversity	
	- Increased abundance of Proteobacteria and decreased abundance of Firmicutes and Bacteroidetes	
	- Significantly increased abundance of *Akkermansia muciniphila*	
	- Four differentially abundant metabolites identified: chenodeoxycholic acid, ursodeoxycholic acid, alpha-linolenic acid (ALA), and 12,13s-epoxy-9z,11,15z-octadecatrienoic acid (12,13-EOTrE)	
Endometriotic mouse model – i.p. injection	- Significant reduction in butyrate concentration in faeces	Chadchan *et al.* (2021)
	- n-butyrate supplementation significantly decreased the size of endometriotic lesions and the number of proliferative cells and macrophages	
Endometriotic mouse model – i.p. injection	- Higher abundance of Firmicutes and lower abundance of Bacteroidetes	Ni *et al.* (2021)
	- Supplementation with ALA restored the abundance of Firmicutes and Bacteroidetes, enhanced the intestinal barrier, and reduced levels of LPS and macrophages	
Endometriotic olive baboon model – surgical	- Significant differences in gut microbiota α- and β-diversities after 3 months, with α-diversity recovering by 15 months	Le *et al.* (2022)
	- Changes in α-diversity positively correlated with circulating Treg populations	
Endometriotic mouse model – surgical	- Six differentially abundant metabolites identified: quinic acid; cytosine; 1-methyl-histidine; *N*^G^,*N*^G^-dimethyl l-arginine; 2-aminoheptanoic acid; and n-acetyl aspartic acid	Chadchan *et al.* (2023)
	- Supplementation with quinic acid resulted in significantly larger endometriotic lesions	
Endometriotic mouse model – i.p. injection	- Significantly lower gut microbiota α-diversity at 28, but not 14, days after induction	Wei *et al.* (2023)
	- Differences in β-diversity at 14 and 28 days	
	- Significantly higher levels of LPS in PF	
	- Injection of β-glucuronidase led to significant increases in: concentration of LPS in PF; number of macrophages; and number and size of endometrial lesions	
B) Clinical studies		
Stage 3/4 endometriosis patients (*n* = 14)	- No difference in gut microbiota α- and β-diversities	Ata *et al.* (2019)
Endometriosis patients (*n* = 35)	- No difference in gut microbiota α- and β-diversities	Perrotta *et al.* (2020)
Endometriosis patients (*n* = 21)	- Significantly reduced gut microbiota α- and β-diversities	Huang *et al.* (2021)
Stage 3/4 endometriosis patients (*n* = 12)	- Non-significantly reduced gut microbiota α-diversity compared to controls	Shan *et al.* (2021)
	- Increased Firmicutes/Bacteroidetes ratio	
	- Higher abundance of *Prevotella*	
	- Higher circulating levels of PGE2 and IL-8	
Endometriosis patients (*n* = 66)	- Significantly reduced gut microbiota α- and β-diversities	Svensson *et al.* (2021)
	- Correlation between *Prevotella* abundance and GI-associated symptoms	
Endometriosis patients (*n* = 35)	- No difference in gut microbiota α- and β-diversities	Wei *et al.* (2023)
	- Significantly higher serum levels of β-glucuronidase	
	- Significantly increased β-glucuronidase expression in endometrial lesions compared to normal endometrium	

Human studies exploring the gut microbiota in endometriosis patients are also limited, with notable inconsistency in findings. For example, whilst some studies found no differences in bacterial diversity in endometriosis patients compared to controls, others identified significant changes (Table 2B). Interestingly, there was a correlation between the abundance of *Prevotella* and GI symptoms, but variations in genetic, dietary, and environmental factors limit interpretation/detection of disease-specific differences, making it difficult to draw robust conclusions.

## The role of the microbiota–gut–brain axis in key symptoms of endometriosis – insights from other relevant conditions

The influence of the MGB axis on the immune system has been investigated in several chronic inflammatory pain conditions. In this section we consider data from studies on conditions and symptoms relevant to endometriosis.

### Pain and inflammation

Studies in animal models have provided evidence to support the two-way connection between pain pathways and the gut microbiota (Table 3). For example, germ-free mice lacking gut bacteria had increased visceral hypersensitivity, which was normalised following microbial re-colonisation (Luczynski *et al.* 2017). The phenotype was found to be transferable by faecal microbiota transfer (FMT) (Yang *et al.* 2019, Lucarini *et al.* 2022), and attenuated by treatment with antibiotics (Aguilera *et al.* 2021, Ding *et al.* 2021), potentially in an inflammasome-dependent manner (Scuderi *et al.* 2020, Aguilera *et al.* 2021). Taken together, these studies support a complex role involving the gut microbiota and immune interactions in pain responses.

**Table 3:** Research studies in animal models investigating the relationship between the gut microbiota, pain, and inflammation.

Model	Findings	Reference
Mouse model of PI-IBS and PI-IBS treated with *Bifidobacterium longum*	- VH was significantly lower in *B. longum*-treated mice compared to PI-IBS.	Gu *et al.* (2016)
	- IL-18 and IL-1β expression were significantly lower in *B. longum*-treated mice compared to PI-IBS.	
	- *B. longum* may inhibit NLRP3 inflammasome.	
MD rat model (male)	- Significantly increased VH but no significant difference in total pain scores.	Hoban *et al.* (2016)
	- More depressive-like behaviours.	
	- Decreased gut microbiota β-diversity.	
Germ-free mice	- Significantly increased VH, which normalised following microbial colonisation.	Luczynski *et al.* (2017)
	- Altered volume of pain-processing brain structures: smaller anterior cingulate cortex and larger periaqueductal grey.	
MS mouse model vs TLR4 KO-MS mouse model	- Increased VH in MS but not in TLR4 KO-MS mice.	Tang *et al.* (2017)
	- LPS-treatment induced VH in MS via TLR4.	
	- VH blocked by inhibition of TLR4 signalling.	
Mouse model of alcohol-induced neuroinflammation (female)	- Neuroinflammation and increased intestinal proinflammatory cytokines attenuated with Ab treatment.	Lowe *et al.* (2018)
	- Ab-treatment increased mRNA expression of some inflammasome components.	
Rat model of spared nerve injury (male)	- Depression-susceptible rats had significantly decreased gut microbiota α-diversity.	Yang *et al.* (2019)
	- FMT to MD-mice transferred a painful phenotype and depression-like behaviours.	
Rat model of IBS	- Inflammasome inhibition reduced NF-ΚB expression and inflammation, and restored IBS-associated tight junction alterations.	Scuderi *et al.* (2020)
Mouse model of inflammasome inhibition*	- Increased abundance of Bifidobacterium in *Casp1* KO compared to WT.	Aguilera *et al.* (2021)
	- Antibiotics reduced immune and inflammatory marker expression in *Casp1* KO but not WT.	
	- Antibiotics reduced pain in WT but not *Casp1* KO.	
Mouse model of neuropathic pain^†^	- Gut microbiota induced pain by influencing pro- and anti-inflammatory T cells.	Ding *et al.* (2021)
	- Pain was attenuated by antibiotics.	
Rat model of colitis (male)	- Unique bacterial profile and increased F/B ratio compared to controls.	Lucarini *et al.* (2022)
	- FMT to healthy rats transferred VH.	
	- FMT recipient rats had increased acetate but decreased butyrate.	
	- FMT recipients had increased plasma IL-6 and TGF-β.	

*Casp1 knock-out; ^†^Chronic-constriction injury of sciatic nerve.

Ab, antibiotic; F/B, firmicutes/bacteroidetes ratio; FMT, faecal microbiota transfer; IBS, irritable bowel syndrome; MD, microbiota-depleted; MS, maternal separation; PI, postinfectious; VF, visceral hypersensitivity.

The potential impact of estrobolome-contributing microbial populations on pain sensitivity has also been demonstrated in a recent study: visceral sensitivity fluctuated throughout the estrous cycle in wild-type mice and increased following a reduction in ovarian steroids as a consequence of ovariectomy. Notably, neither of these effects were seen in germ-free mice, suggesting one mechanism of microbial influence on pain occurs in an oestrogen-dependent manner (Tramullas *et al.* 2021).

### Symptoms associated with the GI system

The potential role of the gut microbiota in the symptomology of IBS has been researched in some depth in both patients and animal models (Table 4). IBS patients are reported to have significant differences in microbial diversity compared to healthy controls. The inverse association between *Akkermansia muciniphila* and pain intensity (Cruz-Aguliar *et al.* 2019) is of interest due to its association with improved intestinal barrier function (Cani & de Vos 2017). FMT has provided further supporting evidence, with transfer of the phenotype from patients to mice, and transfer from healthy donors to patients reducing symptoms and re-diversifying the gut microbiota. To date there are no reports of trials using FMT to treat endometriosis patients.

**Table 4:** Research studies investigating the relationship between the gut microbiota and gastrointestinal symptoms.

Model/cohort	Findings	Reference
IBS patients	- Microbial signatures clustered into two groups: normal microbiota vs. increased F/B ratio.	Jeffery *et al.* (2012)
	- IBS with normal microbiota were more likely to have depression.	
	- Suggests potential differing triggers for IBS-like symptoms.	
IBS patients and MD mouse model (male)	- FMT from IBS patients to MD-mice transferred phenotype.	Ge *et al.* (2017)
	- SCFAs and secondary bile acids were subsequently decreased in recipient mice.	
IBS patients and GF mouse model	- FMT from IBS patients to GF-mice transferred phenotype including intestinal barrier dysfunction, innate immune activation, and anxiety-like behaviour.	De Palma *et al.* (2017)
IBS patients and healthy donor	- FMT from healthy donor to IBS patients reduced abdominal pain symptoms and increased α- and β-diversities.	Cruz-Aguliar *et al.* (2019)
	- Patients with microbiota mostly like the donor had the greatest reduction in pain.	
	- Abundance of *Akkermansia muciniphila* inversely correlated with pain intensity.	
Review – multiple cohorts	- Potential influence of *Lactobacillaceae* and *Bacteroides* metabolites on inflammation and bloating.	Pittayanon *et al.* (2019)
IBS patients	- Significant differences in microbiome and metabolome profiles compared to controls.	Jeffery *et al.* (2020)
	- Faecal metabolomes could differentiate IBS patients with and without bile acid malabsorption.	
	- Decreased bacterial diversity.	
Meta-analysis of IBS patients	- FMT from healthy donors significantly decreased IBS symptoms and improved QoL.	Wang *et al.* (2023)
Meta-analysis of IBS patients	- FMT from healthy donors significantly decreased IBS symptoms but decreased QoL.	Halkjær *et al.* (2023)
Review of IBS patients	- Increased F/B ratio in IBS.	Shaikh *et al.* (2023)
	- No specific microbial signature.	

FMT, faecal microbiota transfer; IBS, irritable bowel syndrome; SCFA, short-chain fatty acid; QoL, quality of life.

### Mood disorders

Comorbidity of GI issues and mood disorders is common. However, clinical evidence to support the role of the MGB axis remains limited. Similar to other areas of gut–brain research, differences in methodology and outcome measures have created challenges when comparing data. Although several studies have identified differences in both α- and β-diversities in the gut microbiota of people with depression and anxiety compared to controls, these findings are not consistent (Simpson *et al.* 2021). Additionally, there were no uniform findings in the differing abundance of specific bacterial species associated with neither depression nor anxiety (Simpson *et al.* 2021).

## Potential symptom-management strategies for endometriosis via manipulation of the gut microbiota

### Dietary modifications

Clinical trials of dietary intervention for disease management are challenging to implement and standardise, with a plethora of variables likely to affect the outcomes (Nap & De Roos 2022). Currently, most research into associations between diet and endometriosis is focussed on risk of disease development, rather than adapting diet for symptom-management (Nap & De Roos 2022). However, there is anecdotal evidence within the endometriosis community for the benefit of dietary modifications as a self-management strategy and some preliminary clinical evidence to support these ideas. Diets (or specific foods) believed to increase bacterial diversity and growth of bacterial species associated with good health are often referred to as ‘prebiotics’, examples include diets rich in fibre and fermented foods (Valdes *et al.* 2018). Health benefits of these diets have been described for various conditions, including IBS (Salmeri *et al.* 2023); however, to date there has been no comprehensive randomised control trial (RCT) in endometriosis patients.

More generally, other research into the impact of Western diet, including the increased consumption of ultra-processed foods, is gaining momentum, with reports of associations with increased low-grade inflammation (Tristan Asensi *et al.* 2023). There is concern that diets high in ultra-processed foods may exacerbate symptoms in those with existing chronic inflammatory conditions, which may also include endometriosis.

#### Current dietary practice in the endometriosis community

Recent surveys investigating the popularity of different diets used by people with endometriosis, and perceived effects on symptoms and QoL, have reported that, although no single dietary intervention appeared to be uniquely effective, many respondents found their chosen modification to be beneficial (Krabbenborg *et al.* 2021, Armour *et al.* 2021). In an Australian survey, 163 respondents had used dietary intervention, 69.0% of whom reported a reduction in the use of pharmaceutical medication (Armour *et al.* 2021). In a Dutch study, 55.5% of the 157 respondents reported nutrition affecting their symptoms and 46.5% were currently following a diet (Krabbenborg *et al.* 2021). These surveys were consistent in finding gluten -free, dairy -free/low lactose, and low fermentable oligosaccharides, disaccharides, monosaccharides and polyols (FODMAPs) as the most popular diets, although both studies are caveated by their relatively small participant numbers and limited geographical reach. The largest survey to date received 1385 responses, predominantly from the UK, of which 52.2% had tried adapting their diet to manage their endometriosis-associated gut symptoms (Deepak Kumar *et al.* 2023). Again, gluten-free was one of the most popular diets; however, only 0.6% were following a low-FODMAP diet, highlighting the strength of a larger dataset.

#### Low-FODMAP diet

FODMAPs are fermentable oligosaccharides, disaccharides, monosaccharides and polyols, which in high doses can cause inflammation and visceral pain (Zhou *et al.* 2017). A low-FODMAP diet is popular for management of IBS, with symptoms shown to improve after three weeks, alongside a reduction in serum levels of proinflammatory cytokines (Hustoft *et al.* 2017). However, long-term use of the low-FODMAP diet may have a negative impact on the gut microbiome (Staudacher 2017). One culprit for inflammatory responses to certain foods is histamine, released by mast cells present in the gut mucosa and further stimulated in a positive feedback loop with oestrogen (Theoharides 2017). Three weeks on a low-FODMAP diet (*n* = 19) was shown to reduce histamine levels eight-fold, in comparison to a high-FODMAP diet (*n* = 18) (McIntosh *et al.* 2017). Histamine mast cells express oestrogen receptors (De Leo *et al.* 2017) and have been implicated in both pathogenesis and pain mechanisms of endometriosis (Kirchhoff *et al.* 2012, Mariuzzi *et al.* 2016), providing a mechanistic link between mast cell activation and intestinal inflammation.

In a study of 160 women, those with both endometriosis and IBS were three-fold more likely to find a low-FODMAP diet effective for improving symptoms, compared to those with IBS alone (Moore *et al.* 2017). This could suggest the cause of IBS-type symptoms frequently reported by endometriosis patients may differ from those with IBS alone and be more receptive to dietary intervention.

### Gluten-free diet

A gluten-free diet is frequently adopted by people with endometriosis; however, there is currently no clinical evidence to support this practice. There has been one retrospective observational study of women with endometriosis who followed a gluten-free diet for 12 months, 75% of whom reported significant pain improvement (Marziali *et al.* 2012). However, 88 of the original 295 participants withdrew within two weeks due to associated abdominal side effects.

### Anti-inflammatory diets

Anti-inflammatory diets, such as the typical Mediterranean diet, consisting of fruit, vegetables, whole grains, and oily fish, with low quantities of dairy and red meat, have been proven to decrease inflammatory markers including IL-6 and C-reactive protein (Tristan Asensi *et al.* 2023). Five months on a Mediterranean diet was found to significantly improve pain in 68 women with endometriosis; however, the study had no control group (Ott *et al.* 2012). A diet high in fermented foods has been shown to increase microbial diversity and decrease inflammatory markers in 18 healthy adults (Wastyk *et al.* 2021).

Signorile *et al.* compared 3 months of an anti-inflammatory dietary supplement, a linseed oil/calcium salt combination, or a placebo, with 30 endometriosis patients in each group (Signorile *et al.* 2018). However, all participants also increased their fibre and omega-3 consumption and cut out soy, aloe, and oats. There was a significant decrease in reported pain symptoms associated with the anti-inflammatory supplement and a significant reduction in serum inflammatory markers (PGE2, CA-125). However, the potential impact of the dietary regime is unclear. A small double-blind RCT compared endometriosis patients taking an eight-week supplement of omega-3 (*n* = 17) versus olive oil (*n* = 16) (Abokhrais *et al.* 2020). Improvements in pelvic pain and QoL scores were seen in both arms; however, there were no significant differences. The use of olive oil in the control arm may explain the results as it has endogenous anti-inflammatory properties (Cicerale *et al.* 2012), (Cicerale *et al.* 2012); therefore, a larger trial is now required with an alternative placebo. Another study also found no benefits of omega-3 over 6 months when comparing fish oil (*n* = 20) to a placebo (*n* = 22) (Nodler *et al.* 2020). Taken together, these data make it difficult to say whether omega-3 supplementation is beneficial.

An earlier study looked at postoperative pain in endometriosis comparing 6 months of hormonal therapy (*n* = 77), dietary therapy (*n* = 35), or placebo (*n* = 110) (Sesti *et al.* 2007). At the 12-month follow-up, all groups reported lower scores for menstrual pain compared to baseline, though these were significantly lower with hormonal, but not dietary, therapy when compared to placebo. On the other hand, following both therapies, non-menstrual pelvic pain was significantly lower than placebo.

### Vitamins

A broad range of vitamins, minerals, and nutritional supplements have been associated with inflammation and immunity, a few of which have been investigated for their potential benefits in endometriosis. Vitamins are important for the normal functioning of the immune system, as well as having antioxidant and anti-inflammatory properties (Carr & Maggini 2017, Lewis *et al.* 2019). The vitamin D receptor is expressed in reproductive tissues, leading to suggestions it may be involved in the aetiology of endometriosis (Barnard *et al.* 2023). A recent online survey with 1385 respondents found 381 (27.5%) took a vitamin D supplement (Deepak Kumar *et al.* 2023).

One study compared 12 weeks of vitamin D (*n* = 19) to a placebo (*n* = 19) and found no difference in reported pelvic pain or dysmenorrhea (Almassinokiani *et al.* 2016). Conversely, another paper reported 12 weeks of vitamin D treatment resulted in significantly decreased pelvic pain, compared to a placebo (*n* = 25 each group) (Mehdizadehkashi *et al.* 2021). A trial by Nodler *et al.*, comparing vitamin D (*n* = 27), fish oil (*n* = 20), and a placebo (*n* = 22), found a significant reduction in ‘worst pain’ associated with vitamin D compared to the other groups (Nodler *et al.* 2020). This study recruited adolescent girls with a mean age of 19.7 – lower than the mean ages of 29.9 and 35.2 in the other two studies. These methodological differences, alongside the small participant numbers, provide a potential explanation for their inconsistency. Other vitamins have also shown promise: women receiving a combination of vitamins C and E (*n* = 30) had significantly lower pain scores for dysmenorrhea, dyspareunia, and CPP after 8 weeks, compared to a placebo (*n* = 30) (Amini *et al.* 2021).

### Curcumin

Curcumin is the active ingredient of turmeric with recognised anti-inflammatory properties (Tabrizi *et al.* 2019). Studies into its use in several health conditions have found reductions in oestrogen concentrations and proinflammatory mediators, as well as inhibition of angiogenesis (Piecuch *et al.* 2022). Following 2 months of daily curcumin supplementation, 33 women with endometriosis experienced significant improvements in pelvic pain, dysmenorrhea, and dyspareunia, with a 48% reduction in the number of participants using nonsteroidal anti-inflammatory drugs (Fadin *et al.* 2020).

### Probiotics

Probiotic treatment, based on ingestion of specific strain(s) of ‘beneficial’ bacteria, is still a relatively new field, with only a few strains available due to culturing and shelf-life constraints. Furthermore, the complex and diverse nature of the gut microbiome means there is still doubt as to whether the added presence of select strains in the form of supplement probiotics can have a significant impact on dysbiosis, considering their unique and sometimes temporary effects on the gut microbiome (Leeming *et al.* 2019).

#### Probiotics in endometriosis

Two RCTs have been conducted using probiotics to treat endometriosis. For 8 weeks, 16 women with endometriosis were given a combination of four different Lactobacillus strains: *Lactobacillus acidophilus; Lactobacillus plantarum; Lactobacillus fermentum;* and* Lactobacillus gasseri*; compared to a placebo (*n* = 16) (Khodaverdi *et al.* 2019). Both groups saw decreases in pain scores for CPP and dyspareunia, and for dysmenorrhea the change was significantly greater in the treatment arm. However, all pain scores had increased by the four-week follow-up which, though they had not reverted to baseline, suggests a potential lack of longevity for the probiotic combination.

In the second study, 29 women were treated with *L. gasseri* for 12 weeks and experienced a significant reduction in pain scores compared to placebo (*n* = 33) (Itoh *et al.* 2011*b*). However, there were no follow-up data and therefore no indication of the long-term impact of the probiotic. The influence of *L. gasseri* on endometriosis has been investigated in rodent models, with an apparent reduction in lesion growth and activation of NK cells (Itoh *et al.* 2011*a*, Uchida and Kobayashi 2013). These data suggest the necessity for additional larger RCTs to investigate the use of *L. gasseri* as a treatment, with a focus on duration of response and the impact of repeated courses of supplementation.

#### Probiotics in other conditions

The use of probiotics as a treatment strategy for IBS has been well documented, with a variety of different strains improving symptom severity, including pain and bloating (Cryan *et al.* 2019, Francavilla *et al.* 2019, Wilmes *et al.* 2021). Studies in animal models have demonstrated the alleviation of visceral pain following probiotic treatment (Zhao *et al.* 2018, Zhang *et al.* 2019, Li *et al.* 2019), though this was not always replicated (Huang *et al.* 2019). One study of 118 IBS patients showed treatment with *L. gasseri* reduced the mean abdominal pain score by 54.2% and attenuated symptoms in 85.0% of participants (Ait Abdellah *et al.* 2022). However, this was not placebo controlled.

Research into the use of probiotics to treat mood disorders provides inconsistent findings (Taylor and Holscher 2020), with varying strains and methodologies used, meaning accurate comparisons are difficult. *Lactobacillus rhamnosus* reduced depression- and anxiety-like behaviour in mice (Bravo *et al.* 2011). Interestingly, in a double-blind RCT, pregnant women treated with *L. rhamnosus* (*n* = 212) reported significantly lower post-partum depression and anxiety scores compared to controls (*n* = 211) (Slykerman *et al.* 2017).

### Antibiotics

Antibiotics have a strong influence on the gut, and their use in early life has been shown to have detrimental effects on the gut microbiota in adulthood (O’Mahony *et al.* 2014). Nonetheless, the preliminary research discussed below raises the potential that symptoms of endometriosis might be treated with antibiotics.

#### Antibiotics in endometriosis

The theoretical benefits for treating endometriosis with antibiotics are multifaceted: perturbation of the gut microbiota could improve pain perception and mood disorders by altering signalling within the MGB axis and even influence disease progression if it blunted the immune response and/or reduced deconjugation of steroids by the estrobolome. Additionally, if bacterial infection is proven to have a causal role in lesion development (Khan *et al.* 2018), then specific antibiotics could provide a defence mechanism against further lesion growth. Some of the studies reviewed below provide support for both these lines of enquiry.

The effect of antibiotics on endometriosis was investigated using an endometriotic mouse model treated with a combination of vancomycin, neomycin, metronidazole, and ampicillin for 3 weeks. Antibiotic-treated mice had smaller lesions with fewer proliferative cells and lower concentrations of proinflammatory cytokines, compared to vehicle-treated controls. However, the gut microbiota in the antibiotic-treated mice had decreased α- and β-diversity, dominated by the phylum Proteobacteria, with negligible abundance of Bacteroidetes and Firmicutes (Chadchan *et al.* 2019). Further analysis of individual treatments with metronidazole or neomycin identified only the former as able to reduce lesion growth (Chadchan *et al.* 2019). The authors suggested this was due to susceptibility of the Bacteroides genus to metronidazole but not neomycin. Importantly, neomycin is a nonabsorbable antibiotic, meaning its influence is restricted to the gut, whereas metronidazole can move into circulation and even interact with the CNS. Therefore, the explanation for these differences could be a result of metronidazole having activities outside the gut.

In a recent study of ovarian endometriosis patients, Muraoka *et al.* reported 64% had *Fusobacterium nucleatum* in their endometrium compared to 7% of controls (*n* = 42 each group) (Muraoka *et al.* 2023). They also used a mouse model of endometriosis combined with injection of *F. nucleatum* and tested the impact of both metronidazole and chloramphenicol. Presence of the bacteria increased lesion size, whereas treatment with antibiotics largely prevented lesion formation and reduced the size of established lesions (Muraoka *et al.* 2023). This appeared to be due to activation of TGF-β1 signalling by the bacteria. Whilst these are new data that must be replicated by others, they do provide strong evidence that antibiotic treatment might be beneficial in some patients.

To date, there has only been one clinical trial investigating the impact of antibiotics on endometriosis patients. In a double-blind RCT, women with stage III/IV endometriosis found 6 months of broad-spectrum antibiotic, clarithromycin (*n* = 129), was no more effective than a placebo (*n* = 160) for reducing pain after surgical removal of lesions (Alborzi *et al.* 2019). Additionally, there was no difference in serum levels of inflammatory biomarkers, including TNF-α, between the two groups.

#### Antibiotics in other conditions

Antibiotics, such as rifaximin, improve symptom severity in patients with IBS (Vicari *et al.* 2017) and decrease visceral pain in animal models (Aguilera *et al.* 2015, Hoban *et al.* 2016). Research into the use of antibiotics for chronic pain with an unknown aetiology is limited, although a recent study into chronic lower back pain found no clinical effect following treatment with amoxicillin (Bråten *et al.* 2019).

## Conclusion and future research

There is increasing, and robust, evidence that the gut microbiota and its metabolites play a key role in the bidirectional signalling pathway between the gut and brain, that can regulate pain, GI symptoms and mood disorders. As these symptoms are common in patients with endometriosis, there is increasing interest in exploring the contribution of the gut microbiota to the manifestation and exacerbation of symptoms, and subsequently whether the use of diet, supplements, probiotics, or antibiotics, all of which may alter the bacterial species in an individual’s microbiome, could be used to improve symptoms and QoL.

Whilst data from endometriosis patients are limited, a large body of work on other chronic conditions has highlighted the impact of the microbiome on mechanisms known to be involved in aetiology, pathogenesis, and symptoms associated with endometriosis. These include immune education and regulation; biosynthesis of bacterial metabolites that interact with immune cells and nerves (enteric and CNS); and steroid metabolism/activation (Cryan *et al.* 2019, Shin *et al.* 2019, Liu *et al.* 2022, Gieryńska *et al.* 2022).

If we are to realise the full potential of the MGB axis as a therapeutic target in endometriosis, it will be essential to develop standardised experimental methodology and to undertake large, well-controlled clinical trials, including careful phenotyping of patients regarding diet, symptoms, and disease stage, complemented by an in-depth analysis of microbial diversity, plus inflammatory and metabolic profiling, allowing comparisons to be made between international cohorts – a technique that has led to breakthroughs in the genetics of endometriosis (Saunders and Horne 2023).

The benefits of large-scale studies on the MGB would be two-fold. They could offer an opportunity to develop a microbial/biomarker profile that could be used to advise patients on personalised self-management strategies, such as the use of diet and probiotics, alongside pharmaceutical and surgical approaches (Fig. 2B). Secondly, if studies on the putative role of bacterial infection in disease progression can be replicated in diverse populations, this could provide a rationale for testing antibiotic treatments for endometriosis. However, these must be approached with caution as antibiotics may also upset the balance of beneficial versus dysbiotic resident gut microbiota.

To summarise, the impact of the gut microbiota on both the aetiology and symptomology of endometriosis is a rapidly expanding field, with some promising avenues for future research focussed on its manipulation to improve patients’ QoL.

## Declaration of interests

FHY, PTKS, and SO have no conflicting interests. AWH is a Co-Editor-in-Chief of *Reproduction and Fertility*. AWH was not involved in the review or editorial process for this paper, on which he is listed as an author. AWH’s institution (The University of Edinburgh) has received payment for consultancy and grant funding from Roche Diagnostics to assist in the early development of a possible blood diagnostic biomarker for endometriosis. AWH’s institution has received payment for consultancy fees from Gesynta and Joii. AWH has received payment for a presentation from Theramex. AWH’s institution has received grant funding from the MRC, NIHR, CSO, and Wellbeing of Women for endometriosis research. AWH is listed as a co-inventor on a UK Patent Application (No. 2217921.2).

## Author contributions

Article conception, FHY, AWH, and PTKS; literature survey and writing, FHY; editing and reviewing, FHY, AWH, PTKS, and SO; supervision, AWH, PTKS, and SO.

## References

[bib1] Abokhrais IM Denison FC Whitaker LHR Saunders PTK Doust A Williams LJ & Horne AW 2020 A two-arm parallel double-blind randomised controlled pilot trial of the efficacy of Omega-3 polyunsaturated fatty acids for the treatment of women with endometriosis-associated pain (PurFECT1). PLoS One 15 e0227695. (10.1371/journal.pone.0227695) 31951599 PMC6968860

[bib2] Aguilera M Cerdà-Cuéllar M & Martínez V 2015 Antibiotic-induced dysbiosis alters host-bacterial interactions and leads to colonic sensory and motor changes in mice. Gut Microbes 6 10–23. (10.4161/19490976.2014.990790) 25531553 PMC4615720

[bib3] Aguilera M Rossini V Hickey A Simnica D Grady F Felice VD Moloney A Pawley L Fanning A Mccarthy L*et al.* 2021 Inflammasome signaling regulates the microbial–neuroimmune axis and visceral pain in mice. International Journal of Molecular Sciences 22 8336. (10.3390/ijms22158336) 34361102 PMC8371481

[bib4] Ait Abdellah S Scanzi J Gal C Martin M Beck M & Ojetti V 2022 Lactobacillus gasseri LA806 supplementation in patients with irritable bowel syndrome: a multicenter study. Journal of Clinical Medicine 11 7446. (10.3390/jcm11247446) 36556059 PMC9787120

[bib5] Alborzi S Poordast T Askary E & Dorniani G 2019 Effects of clarithromycin on inflammatory markers and clinical manifestations in postsurgical follow-up of patients with endometriosis: a double-blinded randomized placebo-controlled clinical trial. Archives of Gynecology and Obstetrics 299 1305–1312. (10.1007/s00404-019-05057-4) 30888478

[bib6] Almassinokiani F Khodaverdi S Solaymani-Dodaran M Akbari P & Pazouki A 2016 Effects of vitamin D on endometriosis-related pain: a double-blind clinical trial. Medical Science Monitor 22 4960–4966. (10.12659/msm.901838) 27986972 PMC5189720

[bib7] Amini L Chekini R Nateghi MR Haghani H Jamialahmadi T Sathyapalan T & Sahebkar A 2021 The effect of combined vitamin C and vitamin E supplementation on oxidative stress markers in women with endometriosis: A randomized, triple-blind placebo-controlled clinical trial. Pain Research and Management 2021 5529741. (10.1155/2021/5529741) 34122682 PMC8172324

[bib8] Aredo JV Heyrana KJ Karp BI Shah JP & Stratton P 2017 Relating chronic pelvic pain and endometriosis to signs of sensitization and myofascial pain and dysfunction. Seminars in Reproductive Medicine 35 88–97. (10.1055/s-0036-1597123) 28049214 PMC5585080

[bib9] Armour M Middleton A Lim S Sinclair J Varjabedian D & Smith CA 2021 Dietary practices of women with endometriosis: a cross-sectional survey. Journal of Alternative and Complementary Medicine 27 771–777. (10.1089/acm.2021.0068) 34161144

[bib10] Arpaia N Campbell C Fan X Dikiy S Van Der Veeken J Deroos P Liu H Cross JR Pfeffer K Coffer PJ, *et al.* 2013 Metabolites produced by commensal bacteria promote peripheral regulatory T-cell generation. Nature 504 451–455. (10.1038/nature12726) 24226773 PMC3869884

[bib11] Asante A & Taylor RN 2011 Endometriosis: the role of neuroangiogenesis. Annual Review of Physiology 73 163–182. (10.1146/annurev-physiol-012110-142158) 21054165

[bib12] As-Sanie S Harris RE Napadow V Kim J Neshewat G Kairys A Williams D Clauw DJ & Schmidt-Wilcke T 2012 Changes in regional gray matter volume in women with chronic pelvic pain: A voxel-based morphometry study. Pain 153 1006–1014. (10.1016/j.pain.2012.01.032) 22387096 PMC3613137

[bib13] As-Sanie S Kim J Schmidt-Wilcke T Sundgren PC Clauw DJ Napadow V & Harris RE 2016 Functional connectivity is associated with altered brain chemistry in women with endometriosis-associated chronic pelvic pain. Journal of Pain 17 1–13. (10.1016/j.jpain.2015.09.008) 26456676 PMC4698023

[bib138] Ata B Yildiz S Turkgeldi E Brocal VP Dinleyici EC Moya A & Urman B. The Endobiota Study: Comparison of Vaginal, Cervical and Gut Microbiota Between Women with Stage 3/4 Endometriosis and Healthy Controls. Scientific Reports 9 2204. (10.1038/s41598-019-39700-6) PMC6379373 30778155

[bib14] Aupetit A Grigioni S Roman H Coëffier M Bréant A Hennetier C & Achamrah N 2022 Association between endometriosis, irritable bowel syndrome and eating disorders: ENDONUT pilot study. Journal of Clinical Medicine 11 5773. (10.3390/jcm11195773) 36233641 PMC9571159

[bib139] Bailey MT & Coe CL 2002 Endometriosis is associated with an altered profile of intestinal microflora in female rhesus monkeys. Human Reproduction 17 1704–1708. (10.1093/humrep/17.7.1704) 12093827

[bib15] Bao A-M & Swaab DF 2019 The human hypothalamus in mood disorders: the HPA axis in the center. IBRO Reports 6 45–53. (10.1016/j.ibror.2018.11.008) 31211281 PMC6562194

[bib16] Barnard ND Holtz DN Schmidt N Kolipaka S Hata E Sutton M Znayenko-Miller T Hazen ND Cobb C & Kahleova H 2023 Nutrition in the prevention and treatment of endometriosis: a review. Frontiers in Nutrition 10 1089891. (10.3389/fnut.2023.1089891) 36875844 PMC9983692

[bib17] Berkley KJ Rapkin AJ & Papka RE 2005 The pains of endometriosis. Science 308 1587–1589. (10.1126/science.1111445) 15947176

[bib18] Boccella S Guida F De Logu F De Gregorio D Mazzitelli M Belardo C Iannotta M Serra N Nassini R Novellis V, *et al.* 2019 Ketones and pain: unexplored role of hydroxyl carboxylic acid receptor type 2 in the pathophysiology of neuropathic pain. FASEB Journal 33 1062–1073. (10.1096/fj.201801033R) 30085883

[bib19] Bråten LCH Rolfsen MP Espeland A Wigemyr M Assmu J Froholdt A Haugen AJ Marchand GH Kristoffersen PM Lutro O, *et al.* 2019 Efficacy of antibiotic treatment in patients with chronic low back pain and Modic changes (the AIM study): double blind, randomised, placebo controlled, multicentre trial. BMJ 367 l5654. (10.1136/bmj.l5654) 31619437 PMC6812614

[bib20] Bravo JA Forsythe P Chew MV Escaravage E Savignac HM Dinan TG Bienenstock J & Cryan JF 2011 Ingestion of Lactobacillus strain regulates emotional behavior and central GABA receptor expression in a mouse via the vagus nerve. Proceedings of the National Academy of Sciences of the United States of America 108 16050–16055. (10.1073/pnas.1102999108) 21876150 PMC3179073

[bib21] Brawn J Morotti M Zondervan KT Becker CM & Vincent K 2014 Central changes associated with chronic pelvic pain and endometriosis. Human Reproduction Update 20 737–747. (10.1093/humupd/dmu025) 24920437 PMC4501205

[bib22] Cani PD & De Vos WM 2017 Next-generation beneficial microbes: the case of Akkermansia muciniphila. Frontiers in Microbiology 8 1765. (10.3389/fmicb.2017.01765) 29018410 PMC5614963

[bib137] Cao Y Jiang C Jia Y Xu D & Yu Y 2020 Letrozole and the Traditional Chinese Medicine, Shaofu Zhuyu Decoction, Reduce Endometriotic Disease Progression in Rats: A Potential Role for Gut Microbiota. Evidence-Based Complementary and Alternative Medicine 2020 3687498. (10.1155/2020/3687498) PMC7387974 32765629

[bib23] Carr AC & Maggini S 2017 Vitamin C and immune function. Nutrients 9 1211. (10.3390/nu9111211) 29099763 PMC5707683

[bib24] Chadchan SB Cheng M Parnell LA Yin Y Schriefer A Mysorekar IU & Kommagani R 2019 Antibiotic therapy with metronidazole reduces endometriosis disease progression in mice: a potential role for gut microbiota. Human Reproduction 34 1106–1116. (10.1093/humrep/dez041) 31037294 PMC6554192

[bib142] Chadchan SB Popli P Ambati CR Tycksen E Han SJ Bulun SE Putluri N Biest SW & Kommagani R 2021 Gut microbiota-derived short-chain fatty acids protect against the progression of endometriosis. Life Science Alliance 30 e202101224. (10.26508/lsa.202101224) PMC8500332 34593556

[bib25] Chadchan SB Naik SK Popli P Talwar C Putluri S Ambati CR Lint MA Kau AL Stallings CL & Kommagani R 2023 Gut microbiota and microbiota-derived metabolites promotes endometriosis. Cell Death Discovery 9 28. (10.1038/s41420-023-01309-0) 36693853 PMC9873805

[bib26] Chiaffarino F Cipriani S Ricci E Mauri PA Esposito G Barretta M Vercellini P & Parazzini F 2021 Endometriosis and irritable bowel syndrome: a systematic review and meta-analysis. Archives of Gynecology and Obstetrics 303 17–25. (10.1007/s00404-020-05797-8) 32949284

[bib27] Cicerale S Lucas LJ & Keast RSJ 2012 Antimicrobial, antioxidant and anti-inflammatory phenolic activities in extra virgin olive oil. Current Opinion in Biotechnology 23 129–135. (10.1016/j.copbio.2011.09.006) 22000808

[bib28] Cruz-Aguliar RM Wantia N Clavel T Vehreschild MJGT Buch T Bajbouj M Haller D Busch D Schmid RM & Stein-Thoeringer CK 2019 An open-labeled study on fecal microbiota transfer in irritable bowel syndrome patients reveals improvement in abdominal pain associated with the relative abundance of *Akkermansia Muciniphila*. Digestion 100 127–138. (10.1159/000494252) 30423561

[bib29] Cryan JF O’riordan KJ Cowan CSM Sandhu KV Bastiaanssen TFS Boehme M Codagnone MG Cussotto S Fulling C Golubeva AV, *et al.* 2019 The microbiota-gut-brain axis. Physiological Reviews 99 1877–2013. (10.1152/physrev.00018.2018) 31460832

[bib30] De Leo B Esnal-Zufiaurre A Collins F Critchley HOD & Saunders PTK 2017 Immunoprofiling of human uterine mast cells identifies three phenotypes and expression of ERβ and glucocorticoid receptor. F1000Research 6 667. (10.12688/f1000research.11432.2) 28620462 PMC5461902

[bib140] De Palma G Lynch MD Lu J Dang VT Deng Y Jury J Umeh G Miranda PM Pigrau Pastor M Sidani Set al. 2017 Transplantation of fecal microbiota from patients with irritable bowel syndrome alters gut function and behavior in recipient mice. Science Translational Medicine 9 eaaf6397. (10.1126/scitranslmed.aaf6397) 28251905

[bib31] Deepak Kumar K Appleby‐Gunnill B & Maslin K 2023 Nutritional practices and dietetic provision in the endometriosis population, with a focus on functional gut symptoms. Journal of Human Nutrition and Dietetics 36 1529–1538. (10.1111/jhn.13158) 36794746

[bib32] Ding W You Z Chen Q Yang L Doheny J Zhou X Li N Wang S Hu K Chen L, *et al.* 2021 Gut microbiota influences neuropathic pain through modulating proinflammatory and anti-inflammatory T cells. Anesthesia and Analgesia 132 1146–1155. (10.1213/ANE.0000000000005155) 32889847

[bib33] Erny D Hrabě De Angelis AL Jaitin D Wieghofer P Staszewski O David E Keren-Shaul H Mahlakoiv T Jakobshagen K Buch T, *et al.* 2015 Host microbiota constantly control maturation and function of microglia in the CNS. Nature Neuroscience 18 965–977. (10.1038/nn.4030) 26030851 PMC5528863

[bib34] Ervin SM Li H Lim L Roberts LR Liang X Mani S & Redinbo MR 2019 Gut microbial β-glucuronidases reactivate estrogens as components of the estrobolome that reactivate estrogens. Journal of Biological Chemistry 294 18586–18599. (10.1074/jbc.RA119.010950) 31636122 PMC6901331

[bib35] Fadin M Nicoletti MC Pellizzato M Accardi M Baietti MG & Fratter A 2020 Effectiveness of the integration of quercetin, turmeric, and N-acetylcysteine in reducing inflammation and pain associated with endometriosis. In-vitro and in-vivo studies. Minerva Ginecologica 72 285–291. (10.23736/S0026-4784.20.04615-8) 32921020

[bib36] Francavilla R Piccolo M Francavilla A Polimeno L Semeraro F Cristofori F Castellaneta S Barone M Indrio F Gobbetti M, *et al.* 2019 Clinical and microbiological effect of a multispecies probiotic supplementation in celiac patients with persistent IBS-type symptoms. Journal of Clinical Gastroenterology 53 e117–e125. (10.1097/MCG.0000000000001023) 29688915 PMC6382041

[bib37] Friggi Sebe Petrelluzzi K Garcia MC Petta CA Ribeiro DA De Oliveira Monteiro NR Céspedes IC & Spadari RC 2012 Physical therapy and psychological intervention normalize cortisol levels and improve vitality in women with endometriosis. Journal of Psychosomatic Obstetrics & Gynecology 33 191–198. (10.3109/0167482X.2012.729625) 23094607

[bib141] Ge X Zhao W Ding C Tian H Xu L Wang H Ni L Jiang J Gong J Zhu Wet al. 2017 Potential role of fecal microbiota from patients with slow transit constipation in the regulation of gastrointestinal motility. Scientific Reports 7 441. (10.1038/s41598-017-00612-y) 28348415 PMC5428802

[bib38] Gete DG Doust J Mortlock S Montgomery G & Mishra GD 2023 Impact of endometriosis on women's health-related quality of life: A national prospective cohort study. Maturitas 174 1–7. (10.1016/j.maturitas.2023.04.272) 37182389

[bib39] Gieryńska M Szulc-Dąbrowska L Struzik J Mielcarska MB & Gregorczyk-Zboroch KP 2022 Integrity of the intestinal barrier: the involvement of epithelial cells and microbiota—A mutual relationship. Animals 12. (10.3390/ani12020145) PMC8772550 35049768

[bib143] Gu QY Zhang J & Feng YC 2016 Role of NLRP3 inflammasome in Bifidobacterium longum-regulated visceral hypersensitivity of postinfectious irritable bowel syndrome. Artificial Cells, Nanomedicine, and Biotechnology 44 1933–1937. (10.3109/21691401.2015.1111238) 26697916

[bib40] Guo R Chen L-H Xing C & Liu T 2019 Pain regulation by gut microbiota: molecular mechanisms and therapeutic potential. British Journal of Anaesthesia 123 637–654. (10.1016/j.bja.2019.07.026) 31551115

[bib144] Halkjær SI Lo B Cold F Højer Christensen A Holster S König J Brummer RJ Aroniadis OC Lahtinen P Holvoet Tet al. 2023 Fecal microbiota transplantation for the treatment of irritable bowel syndrome: A systematic review and meta-analysis. World Journal of Gastroenterology 29 3185–3202. (10.3748/wjg.v29.i20.3185) 37346153 PMC10280798

[bib145] Hantschel J Weis S Schäfer KH Menger MD Kohl M Egert M & Laschke MW 2019 Effect of endometriosis on the fecal bacteriota composition of mice during the acute phase of lesion formation. PLoS One 14 e0226835. (10.1371/journal.pone.0226835) 31887116 PMC6936831

[bib41] He W Liu X Zhang Y & Guo S-W 2010 Generalized hyperalgesia in women with endometriosis and its resolution following a successful surgery. Reproductive Sciences 17 1099–1111. (10.1177/1933719110381927) 20923950

[bib42] Herington JL Bruner-Tran KL Lucas JA & Osteen KG 2011 Immune interactions in endometriosis. Expert Review of Clinical Immunology 7 611–626. (10.1586/eci.11.53) 21895474 PMC3204940

[bib43] Hickey M Missmer SA & Horne AW 2020 Reclassifying endometriosis as a syndrome would benefit patient care. BMJ Opinion. (https://blogs.bmj.com/bmj/2020/08/11/reclassifying-endometriosis-as-a-syndrome-would-benefit-patient-care/)

[bib44] Hoban AE Moloney RD Golubeva AV Mcvey Neufeld KA O’sullivan O Patterson E Stanton C Dinan TG Clarke G & Cryan JF 2016 Behavioural and neurochemical consequences of chronic gut microbiota depletion during adulthood in the rat. Neuroscience 339 463–477. (10.1016/j.neuroscience.2016.10.003) 27742460

[bib45] Horne AW & Missmer SA 2022 Pathophysiology, diagnosis, and management of endometriosis. BMJ 379 e070750. (10.1136/bmj-2022-070750) 36375827

[bib46] Horne AW Saunders PTK Abokhrais IM Hogg L & Endometriosis Priority Setting Partnership Steering Group (appendix) 2017 Top ten endometriosis research priorities in the UK and Ireland. Lancet 389 2191–2192. (10.1016/S0140-6736(1731344-2) 28528751

[bib47] Huang J Zhang C Wang J Guo Q & Zou W 2019 Oral Lactobacillus reuteri LR06 or Bifidobacterium BL5b supplement do not produce analgesic effects on neuropathic and inflammatory pain in rats. Brain and Behavior 9 e01260. (10.1002/brb3.1260) 30839179 PMC6456777

[bib147] Huang L Liu B Liu Z Feng W Liu M Wang Y Peng D Fu X Zhu H Cui Zet al. 2021 Gut Microbiota Exceeds Cervical Microbiota for Early Diagnosis of Endometriosis. Frontiers in Cellular and Infection Microbiology 11 788836. (10.3389/fcimb.2021.788836) 34950610 PMC8688745

[bib48] Hustoft TN Hausken T Ystad SO Valeur J Brokstad K Hatlebakk JG & Lied GA 2017 Effects of varying dietary content of fermentable short-chain carbohydrates on symptoms, fecal microenvironment, and cytokine profiles in patients with irritable bowel syndrome. Neurogastroenterology and Motility 29 e12969. (10.1111/nmo.12969) 27747984

[bib49] Itoh H Sashihara T Hosono A Kaminogawa S & Uchida M 2011a Lactobacillus gasseri OLL2809 inhibits development of ectopic endometrial cell in peritoneal cavity via activation of NK cells in a murine endometriosis model. Cytotechnology 63 205–210. (10.1007/s10616-011-9343-z) 21409454 PMC3080482

[bib50] Itoh H Uchida M Sashihara T Ji Z-S Li J Tang Q Ni S Song L & Kaminogawa S 2011b Lactobacillus gasseri OLL2809 is effective especially on the menstrual pain and dysmenorrhea in endometriosis patients: randomized, double-blind, placebo-controlled study. Cytotechnology 63 153–161. (10.1007/s10616-010-9326-5) 21153437 PMC3080472

[bib149] Jeffery IB O'Toole PW Öhman L Claesson MJ Deane J Quigley EM & Simrén M 2012 An irritable bowel syndrome subtype defined by species-specific alterations in faecal microbiota. Gut 61 997–1006. (10.1136/gutjnl-2011-301501) 22180058

[bib150] Jeffery IB Das A O'Herlihy E Coughlan S Cisek K Moore M Bradley F Carty T Pradhan M Dwibedi Cet al. 2020 Differences in Fecal Microbiomes and Metabolomes of People With vs Without Irritable Bowel Syndrome and Bile Acid Malabsorption. Gastroenterology 158 1016–1028.e8. (10.1053/j.gastro.2019.11.301) 31843589

[bib51] Jess T Frisch M Jørgensen KT Pedersen BV & Nielsen NM 2012 Increased risk of inflammatory bowel disease in women with endometriosis: a nationwide Danish cohort study. Gut 61 1279–1283. (10.1136/gutjnl-2011-301095) 22184069

[bib52] Kawai T & Akira S 2010 The role of pattern-recognition receptors in innate immunity: update on toll-like receptors. Nature Immunology 11 373–384. (10.1038/ni.1863) 20404851

[bib53] Kaya S Hermans L Willems T Roussel N & Meeus M 2013 Central sensitization in urogynecological chronic pelvic pain: a systematic literature review. Pain Physician 16 291–308. 23877446

[bib54] Khan KN Fujishita A Hiraki K Kitajima M Nakashima M Fushiki S & Kitawaki J 2018 Bacterial contamination hypothesis: a new concept in endometriosis. Reproductive Medicine and Biology 17 125–133. (10.1002/rmb2.12083) 29692669 PMC5902457

[bib55] Khodaverdi S Mohammadbeigi R Khaledi M Mesdaghinia L Sharifzadeh F Nasiripour S & Gorginzadeh M 2019 Beneficial effects of oral Lactobacillus on pain severity in WomenSuffering from endometriosis: a pilot placebo-controlled randomized clinical trial. International Journal of Fertility and Sterility 13 178–183. (10.22074/ijfs.2019.5584) 31310070 PMC6642422

[bib56] Kirchhoff D Kaulfuss S Fuhrmann U Maurer M & Zollner TM 2012 Mast cells in endometriosis: guilty or innocent bystanders? Expert Opinion on Therapeutic Targets 16 237–241. (10.1517/14728222.2012.661415) 22332753

[bib57] Krabbenborg I De Roos N Van Der Grinten P & Nap A 2021 Diet quality and perceived effects of dietary changes in Dutch endometriosis patients: an observational study. Reproductive Biomedicine Online 43 952–961. (10.1016/j.rbmo.2021.07.011) 34493462

[bib58] Kuehl LK Michaux GP Richter S Schächinger H & Anton F 2010 Increased basal mechanical pain sensitivity but decreased perceptual wind-up in a human model of relative hypocortisolism. Pain 149 539–546. (10.1016/j.pain.2010.03.026) 20381248

[bib59] Kwa M Plottel CS Blaser MJ & Adams S 2016 The intestinal microbiome and estrogen receptor–positive female breast cancer. Journal of the National Cancer Institute 108. (10.1093/jnci/djw029) PMC5017946 27107051

[bib146] Le N Cregger M Fazleabas A & Braundmeier-Fleming A 2022 Effects of endometriosis on immunity and mucosal microbial community dynamics in female olive baboons. Scientific Reports 12 1590. (10.1038/s41598-022-05499-y) 35102185 PMC8803974

[bib60] Leeming ER Johnson AJ Spector TD & Roy CIL 2019 Effect of diet on the gut microbiota: rethinking intervention duration. Nutrients 11 2862. (10.3390/nu11122862) 31766592 PMC6950569

[bib61] Lewis ED Meydani SN & Wu D 2019 Regulatory role of vitamin E in the immune system and inflammation. IUBMB Life 71 487–494. (10.1002/iub.1976) 30501009 PMC7011499

[bib63] Li Y-J Dai C & Jiang M 2019 Mechanisms of probiotic VSL#3 in a rat model of visceral hypersensitivity involves the mast cell-PAR2-TRPV1 pathway. Digestive Diseases and Sciences 64 1182–1192. (10.1007/s10620-018-5416-6) 30560330

[bib62] Li S Hua D Wang Q Yang L Wang X Luo A & Yang C 2020 The role of bacteria and its derived metabolites in chronic pain and depression: recent findings and research progress. International Journal of Neuropsychopharmacology 23 26–41. (10.1093/ijnp/pyz061) 31760425 PMC7064053

[bib64] Liu J Liu X Duan K Zhang Y & Guo S-W 2012 The expression and functionality of transient receptor potential vanilloid 1 in ovarian endometriomas. Reproductive Sciences 19 1110–1124. (10.1177/1933719112443876) 22556011

[bib65] Liu J Tan Y Cheng H Zhang D Feng W & Peng C 2022 Functions of gut microbiota metabolites, current status and future perspectives. Aging and Disease 13 1106–1126. (10.14336/AD.2022.0104) 35855347 PMC9286904

[bib148] Lowe PP Gyongyosi B Satishchandran A Iracheta-Vellve A Cho Y Ambade A & Szabo G 2018 Reduced gut microbiome protects from alcohol-induced neuroinflammation and alters intestinal and brain inflammasome expression. Journal of Neuroinflammation 15 298. (10.1186/s12974-018-1328-9) 30368255 PMC6203993

[bib66] Lucarini E Di Pilato V Parisio C Micheli L Toti A Pacini A Bartolucci G Baldi S Niccolai E Amedei A, *et al.* 2022 Visceral sensitivity modulation by faecal microbiota transplantation: the active role of gut bacteria in pain persistence. Pain 163 861–877. (10.1097/j.pain.0000000000002438) 34393197 PMC9009324

[bib67] Luczynski P Tramullas M Viola M Shanahan F Clarke G O'mahony S Dinan TG & Cryan JF 2014 Microbiota regulates visceral pain in the mouse. eLife 6. (10.7554/eLife.25887) PMC5478269 28629511

[bib68] Mariuzzi L Domenis R Orsaria M Marzinotto S Londero AP Bulfoni M Candotti V Zanello A Ballico M Mimmi MC, *et al.* 2016 Functional expression of aryl hydrocarbon receptor on mast cells populating human endometriotic tissues. Laboratory Investigation 96 959–971. (10.1038/labinvest.2016.74) 27348627 PMC5008463

[bib69] Maroun P Cooper MJW Reid GD & Keirse MJNC 2009 Relevance of gastrointestinal symptoms in endometriosis. Australian and New Zealand Journal of Obstetrics and Gynaecology 49 411–414. (10.1111/j.1479-828X.2009.01030.x) 19694698

[bib70] Marziali M Venza M Lazzaro S Lazzaro A Micossi C & Stolfi VM 2012 Gluten-free diet: a new strategy for management of painful endometriosis related symptoms? Minerva Chirurgica 67 499–504. 23334113

[bib71] McIntosh K Reed DE Schneider T Dang F Keshteli AH De Palma G Madsen K Bercik P & Vanner S 2017 FODMAPs alter symptoms and the metabolome of patients with IBS: a randomised controlled trial. Gut 66 1241–1251. (10.1136/gutjnl-2015-311339) 26976734

[bib72] Mehdizadehkashi A Rokhgireh S Tahermanesh K Eslahi N Minaeian S & Samimi M 2021 The effect of vitamin D supplementation on clinical symptoms and metabolic profiles in patients with endometriosis. Gynecological Endocrinology 37 640–645. (10.1080/09513590.2021.1878138) 33508990

[bib73] Moore JS Gibson PR Perry RE & Burgell RE 2017 Endometriosis in patients with irritable bowel syndrome: specific symptomatic and demographic profile, and response to the low FODMAP diet. Australian and New Zealand Journal of Obstetrics and Gynaecology 57 201–205. (10.1111/ajo.12594) 28303579

[bib74] Muraoka A Suzuki M Hamaguchi T Watanabe S Iijima K Murofushi Y Shinjo K Osuka S Hariyama Y Ito M, *et al.* 2023 Fusobacterium infection facilitates the development of endometriosis through the phenotypic transition of endometrial fibroblasts. Science Translational Medicine 15 eadd1531. (10.1126/scitranslmed.add1531) 37315109

[bib75] Nap A & De Roos N 2022 Endometriosis and the effects of dietary interventions: what are we looking for? Reproduction and Fertility 3 C14–C22. (10.1530/RAF-21-0110) 35814941 PMC9259892

[bib76] Neziri AY Haesler S Petersen-Felix S Müller M Arendt-Nielsen L Manresa JB Andersen OK & Curatolo M 2010 Generalized expansion of nociceptive reflex receptive fields in chronic pain patients. Pain 151 798–805. (10.1016/j.pain.2010.09.017) 20926191

[bib153] Ni Z Sun S Bi Y Ding J Cheng W Yu J Zhou L Li M & Yu C 2020 Correlation of fecal metabolomics and gut microbiota in mice with endometriosis. American Journal of Reproductive Immunology 84 e13307. (10.1111/aji.13307) 32681566

[bib77] Ni Z Ding J Zhao Q Cheng W Yu J Zhou L Sun S & Yu C 2021 Alpha‐linolenic acid regulates the gut microbiota and the inflammatory environment in a mouse model of endometriosis. American Journal of Reproductive Immunology 86 e13471. (10.1111/aji.13471) 34022075

[bib78] Nodler JL Divasta AD Vitonis AF Karevicius S Malsch M Sarda V Fadayomi A Harris HR & Missmer SA 2020 Supplementation with vitamin D or ω-3 fatty acids in adolescent girls and young women with endometriosis (SAGE): a double-blind, randomized, placebo-controlled trial. American Journal of Clinical Nutrition 112 229–236. (10.1093/ajcn/nqaa096) 32453393 PMC7326593

[bib81] O’Mahony SM, Felice VD Nally K Savignac HM Claesson MJ Scully P Woznicki J Hyland NP Shanahan F Quigley EM, *et al.* 2014 Disturbance of the gut microbiota in early-life selectively affects visceral pain in adulthood without impacting cognitive or anxiety-related behaviors in male rats. Neuroscience 277 885–901. (10.1016/j.neuroscience.2014.07.054) 25088912

[bib79] O’Mahony SM Clarke G Borre YE Dinan TG & Cryan JF 2015 Serotonin, tryptophan metabolism and the brain-gut-microbiome axis. Behavioural Brain Research 277 32–48. (10.1016/j.bbr.2014.07.027) 25078296

[bib80] O'Mahony SM Dinan TG & Cryan JF 2017 The gut microbiota as a key regulator of visceral pain. Pain 158(Supplement 1) S19–S28. (10.1097/j.pain.0000000000000779) 27918315

[bib82] Ortiz R Gemmill JAL Sinaii N Stegmann B Khachikyan I Chrousos G Segars J & Stratton P 2020 Hypothalamic-pituitary-adrenal axis responses in women with endometriosis-related chronic pelvic pain. Reproductive Sciences 27 1839–1847. (10.1007/s43032-020-00201-x) 32572832 PMC12452883

[bib83] Ott J Nouri K Hrebacka D Gutschelhofer S Huber J & Wenzl R 2012 Endometriosis and nutrition – recommending a Mediterranean diet decreases endometriosisassociated pain: an experimental observational study. Journal of Aging Research and Clinical Practice 1 162–166.

[bib84] Oyola MG & Handa RJ 2017 Hypothalamic–pituitary–adrenal and hypothalamic–pituitary–gonadal axes: sex differences in regulation of stress responsivity. Stress 20 476–494. (10.1080/10253890.2017.1369523) 28859530 PMC5815295

[bib151] Perrotta AR Borrelli GM Martins CO Kallas EG Sanabani SS Griffith LG Alm EJ & Abrao MS 2020 The Vaginal Microbiome as a Tool to Predict rASRM Stage of Disease in Endometriosis: a Pilot Study. Reproductive Sciences 27 1064–1073. (10.1007/s43032-019-00113-5) 32046455 PMC7539818

[bib85] Piecuch M Garbicz J Waliczek M Malinowska-Borowska J & Rozentryt P 2022 I am the 1 in 10—what should I eat? A research review of nutrition in endometriosis. Nutrients 14 5283. (10.3390/nu14245283) 36558442 PMC9783589

[bib152] Pittayanon R Lau JT Yuan Y Leontiadis GI Tse F Surette M Moayyedi P 2019 Gut Microbiota in Patients With Irritable Bowel Syndrome-A Systematic Review. Gastroenterology 157 97–108. (10.1053/j.gastro.2019.03.049) 30940523

[bib86] Rea K O'mahony S Dinan TG & Cryan JF 2019 Pain bugs: gut microbiota and pain disorders. Current Opinion in Physiology 11 97–102. (10.1016/j.cophys.2019.10.001)

[bib87] Rusch JA Layden BT & Dugas LR 2023 Signalling cognition: the gut microbiota and hypothalamic-pituitary-adrenal axis. Frontiers in Endocrinology (Lausanne) 14 1130689–1130689. (10.3389/fendo.2023.1130689) PMC10316519 37404311

[bib88] Salmeri N Sinagra E Dolci C Buzzaccarini G Sozzi G Sutera M Candiani M Ungaro F Massimino L Danese S, *et al.* 2023 Microbiota in irritable bowel syndrome and endometriosis: birds of a feather flock together—a review. Microorganisms 11 2089. (10.3390/microorganisms11082089) 37630649 PMC10458414

[bib89] Saraswat L Ayansina D Cooper KG Bhattacharya S Horne AW & Bhattacharya S 2018 Impact of endometriosis on risk of further gynaecological surgery and cancer: a national cohort study. BJOG 125 64–72. (10.1111/1471-0528.14793) 28952173

[bib90] Saunders PTK 2022 Insights from genomic studies on the role of sex steroids in the aetiology of endometriosis. Reproduction and Fertility 3 R51–R65. (10.1530/RAF-21-0078) 35514537 PMC9066947

[bib91] Saunders PTK & Horne AW 2023 Genetic analysis confirms a link between gastrointestinal disorders and endometriosis. Cell Reports. Medicine 4 101288. (10.1016/j.xcrm.2023.101288) 37992677 PMC10694734

[bib92] Saunders PTK & Horne AWT 2021 Endometriosis: etiology, pathobiology, and therapeutic prospects. Cell 184 2807–2824. (10.1016/j.cell.2021.04.041) 34048704

[bib93] Scuderi SA Casili G Lanza M Filippone A Paterniti I Esposito E & Campolo M 2020 Modulation of NLRP3 inflammasome attenuated inflammatory response associated to diarrhea-predominant irritable bowel syndrome. Biomedicines 8 519. (10.3390/biomedicines8110519) 33233503 PMC7699594

[bib94] Sesti F Pietropolli A Capozzolo T Broccoli P Pierangeli S Bollea MR & Piccione E 2007 Hormonal suppression treatment or dietary therapy versus placebo in the control of painful symptoms after conservative surgery for endometriosis stage III–IV. A randomized comparative trial. Fertility and Sterility 88 1541–1547. (10.1016/j.fertnstert.2007.01.053) 17434511

[bib154] Shaikh SD Sun N Canakis A Park WY & Weber HC 2023 Irritable Bowel Syndrome and the Gut Microbiome: A Comprehensive Review. Journal of Clinical Medicine 12 2558. (10.3390/jcm12072558) 37048642 PMC10095554

[bib155] Shan J Ni Z Cheng W Zhou L Zhai D Sun S & Yu C 2021 Gut microbiota imbalance and its correlations with hormone and inflammatory factors in patients with stage 3/4 endometriosis. Archives of Gynecology and Obstetrics 304 1363–1373. (10.1007/s00404-021-06057-z) 33839907

[bib95] Shin J-H Park Y-H Sim M Kim S-A Joung H & Shin D-M 2019 Serum level of sex steroid hormone is associated with diversity and profiles of human gut microbiome. Research in Microbiology 170 192–201. (10.1016/j.resmic.2019.03.003) 30940469

[bib96] Signorile PG Viceconte R & Baldi A 2018 Novel dietary supplement association reduces symptoms in endometriosis patients. Journal of Cellular Physiology 233 5920–5925. (10.1002/jcp.26401) 29243819

[bib97] Simpson CA Diaz-Arteche C Eliby D Schwartz OS Simmons JG & Cowan CSM 2021 The gut microbiota in anxiety and depression – A systematic review. Clinical Psychology Review 83 101943. (10.1016/j.cpr.2020.101943) 33271426

[bib98] Slykerman RF Hood F Wickens K Thompson JMD Barthow C Murphy R Kang J Rowden J Stone P Crane J, *et al.* 2017 Effect of Lactobacillus rhamnosus HN001 in pregnancy on postpartum symptoms of depression and anxiety: A randomised double-blind placebo-controlled trial. EBiomedicine 24 159–165. (10.1016/j.ebiom.2017.09.013) 28943228 PMC5652021

[bib99] Sommer F & Bäckhed F 2013 The gut microbiota — masters of host development and physiology. Nature Reviews. Microbiology 11 227–238. (10.1038/nrmicro2974) 23435359

[bib100] Spichak S Guzzetta KE O’leary OF Clarke G Dinan TG & Cryan JF 2018 Without a bug’s life: Germ-free rodents to interrogate microbiota-gut-neuroimmune interactions. Drug Discovery Today: Disease Models 28 79–93. (10.1016/j.ddmod.2019.08.002)

[bib101] Staudacher HM 2017 Nutritional, microbiological and psychosocial implications of the low FODMAP diet. Journal of Gastroenterology and Hepatology 32(Supplement 1) 16–19. (10.1111/jgh.13688) 28244658

[bib156] Svensson A Brunkwall L Roth B Orho-Melander M & Ohlsson B 2021 Associations Between Endometriosis and Gut Microbiota. Reproductive Sciences 28 2367–2377. (10.1007/s43032-021-00506-5) 33660232 PMC8289757

[bib102] Symons LK Miller JE Kay VR Marks RM Liblik K Koti M & Tayade C 2018 The Immunopathophysiology of endometriosis. Trends in Molecular Medicine 24 748–762. (10.1016/j.molmed.2018.07.004) 30054239

[bib103] Tabrizi R Vakili S Akbari M Mirhosseini N Lankarani KB Rahimi M Mobini M Jafarnejad S Vahedpoor Z & Asemi Z 2019 The effects of curcumin-containing supplements on biomarkers of inflammation and oxidative stress: a systematic review and meta-analysis of randomized controlled trials. Phytotherapy Research 33 253–262. (10.1002/ptr.6226) 30402990

[bib104] Talwar C Singh V & Kommagani R 2022 The gut microbiota: a double-edged sword in endometriosis. Biology of Reproduction 107 881–901. (10.1093/biolre/ioac147) 35878972 PMC9562115

[bib105] Tan J Mckenzie C Potamitis M Thorburn AN Mackay CR & Macia L 2014 The role of short-chain fatty acids in health and disease. Advances in Immunology 121 91–119. (10.1016/B978-0-12-800100-4.00003-9) 24388214

[bib157] Tang HL Zhang G Ji NN Du L Chen BB Hua R & Zhang YM 2017 Toll-Like Receptor 4 in Paraventricular Nucleus Mediates Visceral Hypersensitivity Induced by Maternal Separation. Frontiers in Pharmacology 8 309. (10.3389/fphar.2017.00309) 28611665 PMC5447361

[bib106] Taylor AM & Holscher HD 2020 A review of dietary and microbial connections to depression, anxiety, and stress. Nutritional Neuroscience 23 237–250. (10.1080/1028415X.2018.1493808) 29985786

[bib107] Tejada MA Antunez C Nunez-Badinez P De Leo B Saunders PT Vincent K Cano A Nagel J & Gomez R 2023 Rodent animal models of endometriosis-associated pain: unmet needs and resources available for improving translational research in endometriosis. International Journal of Molecular Sciences 24 2422. (10.3390/ijms24032422) 36768741 PMC9917069

[bib108] Theoharides TC 2017 Neuroendocrinology of mast cells: challenges and controversies. Experimental Dermatology 26 751–759. (10.1111/exd.13288) 28094875

[bib109] Tokushige N Russell P Black K Barrera H Dubinovsky S Markham R & Fraser IS 2010 Nerve fibers in ovarian endometriomas. Fertility and Sterility 94 1944–1947. (10.1016/j.fertnstert.2009.12.074) 20153469

[bib110] Tramullas M Collins JM Fitzgerald P Dinan TG O’ Mahony SM & Cryan JF 2021 Estrous cycle and ovariectomy-induced changes in visceral pain are microbiota-dependent. iScience 24 102850. (10.1016/j.isci.2021.102850) 34381975 PMC8333168

[bib111] Tristan Asensi M Napoletano A Sofi F & Dinu M 2023 Low-grade inflammation and ultra-processed foods consumption: a review. Nutrients 15 1546. (10.3390/nu15061546) 36986276 PMC10058108

[bib112] Tu C-H Niddam DM Chao H-T Liu R-S Hwang R-J Yeh T-C & Hsieh J-C 2009 Abnormal cerebral metabolism during menstrual pain in primary dysmenorrhea. NeuroImage 47 28–35. (10.1016/j.neuroimage.2009.03.080) 19362153

[bib114] Uchida M & Kobayashi O 2013 Effects of Lactobacillus gasseri OLL2809 on the induced endometriosis in rats. Bioscience, Biotechnology, and Biochemistry 77 1879–1881. (10.1271/bbb.130319) 24018664

[bib115] Ustianowska K Ustianowski Ł Machaj F Gorący A Rosik J Szostak B Szostak J & Pawlik A 2022 The role of the human microbiome in the pathogenesis of pain. International Journal of Molecular Sciences 23 13267. (10.3390/ijms232113267) 36362056 PMC9659276

[bib116] Valdes AM Walter J Segal E & Spector TD 2018 Role of the gut microbiota in nutrition and health. BMJ 361 36–44. (10.1136/bmj.k2179) PMC6000740 29899036

[bib117] Vercellini P Fedele L Aimi G Pietropaolo G Consonni D & Crosignani PG 2007 Association between endometriosis stage, lesion type, patient characteristics and severity of pelvic pain symptoms: a multivariate analysis of over 1000 patients. Human Reproduction 22 266–271. (10.1093/humrep/del339) 16936305

[bib118] Vicari E Salemi M Sidoti G Malaguarnera M & Castiglione R 2017 Symptom severity following Rifaximin and the probiotic VSL#3 in patients with chronic pelvic pain syndrome (due to inflammatory prostatitis) plus irritable bowel syndrome. Nutrients 9 1208. (10.3390/nu9111208) 29099760 PMC5707680

[bib119] Vincent K Warnaby C Stagg CJ Moore J Kennedy S & Tracey I 2011 Dysmenorrhoea is associated with central changes in otherwise healthy women. Pain 152 1966–1975. (10.1016/j.pain.2011.03.029) 21524851

[bib120] Vinolo MAR Rodrigues HG Nachbar RT & Curi R 2011 Regulation of inflammation by short chain fatty acids. Nutrients 3 858–876. (10.3390/nu3100858) 22254083 PMC3257741

[bib121] Wagner BD Grunwald GK Zerbe GO Mikulich-Gilbertson SK Robertson CE Zemanick ET & Harris JK 2018 On the use of diversity measures in longitudinal sequencing studies of microbial communities. Frontiers in Microbiology 9 1037. (10.3389/fmicb.2018.01037) 29872428 PMC5972327

[bib122] Wang Y & Kasper LH 2014 The role of microbiome in central nervous system disorders. Brain, Behavior, and Immunity 38 1–12. (10.1016/j.bbi.2013.12.015) 24370461 PMC4062078

[bib158] Wang M Xie X Zhao S Ma X Wang Z & Zhang Y 2023 Fecal microbiota transplantation for irritable bowel syndrome: a systematic review and meta-analysis of randomized controlled trials. Frontiers in Immunology 14 1136343. (10.3389/fimmu.2023.1136343) 37275867 PMC10234428

[bib123] Wastyk HC Fragiadakis GK Perelman D Dahan D Merrill BD Yu FB Topf M Gonzalez CG Van Treuren W Han S, *et al.* 2021 Gut-microbiota-targeted diets modulate human immune status. Cell 184 4137–4153.e14. (10.1016/j.cell.2021.06.019) 34256014 PMC9020749

[bib124] Wei Y Tan H Yang R Yang F Liu D Huang B Ouyang L Lei S Wang Z Jiang S, *et al.* 2023 Gut dysbiosis-derived β-glucuronidase promotes the development of endometriosis. Fertility and Sterility 120 682–694. (10.1016/j.fertnstert.2023.03.032) 37178109

[bib159] Weiwei H Xishi L Yuqiu Z & Guo S-W 2010 Endometriosis and its resolution following a successfull surgery. Reproductive Sciences 17 1099–1111. (10.1177/1933719110381927) 20923950

[bib125] Wilmes L Collins JM O'riordan KJ O’mahony SM Cryan JF & Clarke G 2021 Of bowels, brain and behavior: A role for the gut microbiota in psychiatric comorbidities in irritable bowel syndrome. Neurogastroenterology and Motility 33 e14095. (10.1111/nmo.14095) 33580895

[bib126] Yang C Fang X Zhan G Huang N Li S Bi J Jiang R Yang L Miao L Zhu B, *et al.* 2019 Key role of gut microbiota in anhedonia-like phenotype in rodents with neuropathic pain. Translational Psychiatry 9 57. (10.1038/s41398-019-0379-8) 30705252 PMC6355832

[bib127] Yang F Wu Y Hockey R Doust J Mishra GD Montgomery GW & Mortlock S 2023 Evidence of shared genetic factors in the etiology of gastrointestinal disorders and endometriosis and clinical implications for disease management. Cell Reports. Medicine 4 101250. (10.1016/j.xcrm.2023.101250) 37909040 PMC10694629

[bib128] Yano M Matsuda A Natsume T Ogawa SY Awaga Y Hayashi I Hama A & Takamatsu H 2019 Pain-related behavior and brain activation in cynomolgus macaques with naturally occurring endometriosis. Human Reproduction 34 469–478. (10.1093/humrep/dey383) 30597044

[bib129] Yovich JL Rowlands PK Lingham S Sillender M & Srinivasan S 2020 Pathogenesis of endometriosis: look no further than John Sampson. Reproductive Biomedicine Online 40 7–11. (10.1016/j.rbmo.2019.10.007) 31836436

[bib160] Yuan M Li D Zhang Z Sun H An M & Wang G 2018 Endometriosis induces gut microbiota alterations in mice. Human Reproduction 33 607–616. (10.1093/humrep/dex372) 29462324

[bib130] Zhang J Song L Wang Y Liu C Zhang L Zhu S Liu S & Duan L 2019 Beneficial effect of butyrate‐producing Lachnospiraceae on stress‐induced visceral hypersensitivity in rats. Journal of Gastroenterology and Hepatology 34 1368–1376. (10.1111/jgh.14536) 30402954 PMC7379616

[bib131] Zhao K Yu L Wang X He Y & Lu B 2018 Clostridium butyricum regulates visceral hypersensitivity of irritable bowel syndrome by inhibiting colonic mucous low grade inflammation through its action on NLRP6. Acta Biochimica et Biophysica Sinica 50 216–223. (10.1093/abbs/gmx138) 29329362

[bib133] Zheng P Zhang W Leng J & Lang J 2019 Research on central sensitization of endometriosis-associated pain: a systematic review of the literature. Journal of Pain Research 12 1447–1456. (10.2147/JPR.S197667) 31190954 PMC6514255

[bib132] Zheng P Jia S Guo D Chen S Zhang W Cheng A Xie W Sun G Leng J & Lang J 2020 Central sensitization-related changes in brain function activity in a rat endometriosis-associated pain model. Journal of Pain Research 13 95–107. (10.2147/JPR.S232313) 32021399 PMC6968808

[bib134] Zhou S-Y Gillilland M Wu X Leelasinjaroen P Zhang G Zhou H Ye B Lu Y & Owyang C 2017 FODMAP diet modulates visceral nociception by lipopolysaccharide-mediated intestinal inflammation and barrier dysfunction. Journal of Clinical Investigation 128 267–280. (10.1172/JCI92390) 29202473 PMC5749529

[bib135] Zondervan KT Becker CM Koga K Missmer SA Taylor RN & Viganò P 2018 Endometriosis. Nature Reviews. Disease Primers 4 9. (10.1038/s41572-018-0008-5) 30026507

[bib136] Zondervan KT Becker CM & Missmer SA 2020 Endometriosis. New England Journal of Medicine 382 1244–1256. (10.1056/NEJMra1810764) 32212520

